# Where does transcription start? 5′-RACE adapted to next-generation sequencing

**DOI:** 10.1093/nar/gkv1328

**Published:** 2015-11-28

**Authors:** Fleur A.D. Leenen, Sara Vernocchi, Oliver E. Hunewald, Stephanie Schmitz, Anne M. Molitor, Claude P. Muller, Jonathan D. Turner

**Affiliations:** 1 Department of Infection and Immunity, Luxembourg Institute of Health, Esch-Sur-Alzette L-4354, Grand-Duchy of Luxembourg; 2 Department of Immunology, Research Institute of Psychobiology, University of Trier, Trier D-54290, Germany

## Abstract

The variability and complexity of the transcription initiation process was examined by adapting RNA ligase-mediated rapid amplification of 5′ cDNA ends (5′-RACE) to Next-Generation Sequencing (NGS). We oligo-labelled 5′-m^7^G-capped mRNA from two genes, the simple mono-exonic *Beta-2-Adrenoceptor (ADRB2R)* and the complex multi-exonic *Glucocorticoid Receptor (GR, NR3C1)*, and detected a variability in TSS location that has received little attention up to now. Transcription was not initiated at a fixed TSS, but from loci of 4 to 10 adjacent nucleotides. Individual TSSs had frequencies from <0.001% to 38.5% of the total gene-specific 5′ m^7^G-capped transcripts. *ADRB2R* used a single locus consisting of 4 adjacent TSSs. Unstimulated, the *GR* used a total of 358 TSSs distributed throughout 38 loci, that were principally in the 5′ UTRs and were spliced using established donor and acceptor sites. Complete demethylation of the epigenetically sensitive *GR* promoter with 5-azacytidine induced one new locus and 127 TSSs, 12 of which were unique. We induced *GR* transcription with dexamethasone and Interferon-γ, adding one new locus and 185 additional TSSs distributed throughout the promoter region. *In-vitro* the TSS microvariability regulated mRNA translation efficiency and the relative abundance of the different *GR* N-terminal protein isoform levels.

## INTRODUCTION

The genome does not only encode mRNA and protein sequences but it contains also the temporal, spatial and quantitative instructions for their expression. This elaborate regulation occurs principally at the transcriptional level, determining both gene expression and transcript diversity. In the simplest case, transcription is initiated from a transcription start site (TSS) after completing the assembly of the competent transcription initiation complex on the associated promoter. Many genes possess a 5′ UTR containing multiple alternative first exons, each with its own alternative promoter as a second level of transcriptional complexity. It has been estimated that 58% of the transcribed genes had multiple promoters (1). The 5′ UTR‘s influence gene expression in a cell- and tissue-specific manner by generating transcriptional variability, i.e. different mRNA variants (2–5). Whilst some alternative 5′ UTR first exons may be similar in length and nucleotide (nt) sequence, e.g. the Pcdh and UGT1 gene clusters (6), most alternative 5′ UTR first exons differ in length and sequence. These complex 5′ UTRs evolved through processes such as gene duplication by recombination, retroposition, intronic deletions, etc. (7–10). Both alternative splicing and alternative transcription initiation are closely linked and give rise to high complex and diverse transcriptomes and proteomes (5,11–15). Coding 5′ UTR first exons generate different mRNA transcript variants and protein isoforms. Although non-coding first exons do not generate protein diversity, they create transcript variability that has significant impact on post-transcriptional gene regulation, including translational efficiency, mRNA processing, stability and export (3,4,6,16,17).

In eukaryotes, most promoters are located within CpG-rich regions, whilst conserved, well defined TATA box based promoters are less frequent (1,18). Ubiquitously expressed genes are primarily associated with CpG islands and variable TSSs, whereas tightly regulated transcripts have TATA box promoters and well-defined TSSs (1). There is now limited evidence that, irrespective of their location, the site at which transcription is initiated may be variable (1). This was observed as a series of TSSs over a very small 4–6 bp region surrounding the principal TSSs (1).

**Figure 1.**
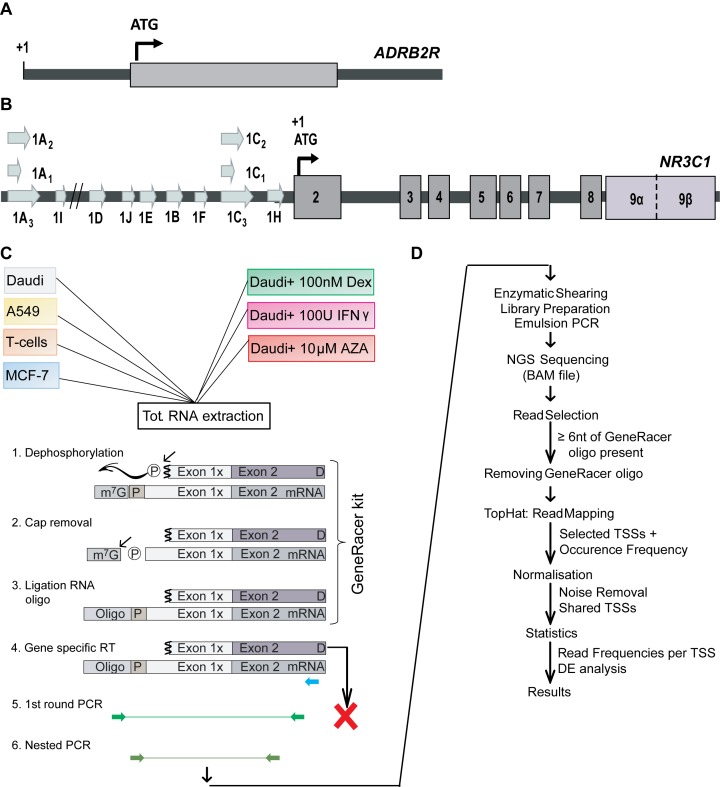
A schematic representation of the *ADRB2R* and *NR3C1* gene structure and the 5′-RACE-Sequencing workflow. (**A**) The *ADRB2R* gene. Nucleotides are numbered with respect to the NCBI reference sequence (NM_000024.5). (**B**) A schematic representation of the *NR3C1* gene, showing the first exons (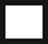) in the distal and proximal (CpG island) promoter; the seven common exons (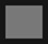); and the two alternative 3′ coding exons (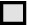). Nucleotides are numbered with respect to the ATG translation initiation codon (+1). (**C**) The experimental workflow from RNA extraction, through the 5′-RACE protocol in order to label the TSSs, the NGS library preparation and the actual sequencing. D, uncapped RNA sequence; M7G mRNA specific 7-methylguanosine cap; 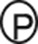
active phosphate; 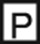
phosphate diesterbond; 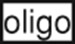
generacer specific oligo. (**D**) The data analysis workflow including read selection, quality control, mapping (TopHat), normalisation, reproducibility and differential expression analysis.

To further investigate the variability of the transcription start sites, two genes with distinct structures and expression profiles were selected. The *Beta-2 Adrenoceptor* (*ADRB2R;* OMIM 109690), is an intronless single exon gene (Figure 1A and Supplementary Data Figure S1A), with no previously identified transcriptional variability, and a uniform, ubiquitous expression according to the literature (19). In comparison, the human *glucocorticoid receptor* gene (*GR*; *NR3C1*, OMIM +138040), located within chromosome 5, has a complex 5′ structure and a highly variable and tightly regulated, but ubiquitous expression (19). The *GR* comprises nine untranslated, alternatively spliced first exons (exon 1A–H) and eight translated exons (exons 2–9), with the translation start site located within exon 2 (Figure 1B and Supplementary Data Figure S1B). All alternative first exons have their own promoter region covering both a CpG island and a distal TATA-like promoter (19–26). They are located either in the distal or the proximal promoter region, 30kb (1A and 1I) and 5kb (1D–J) upstream of the translation start site respectively. The latter are contained in a highly conserved 3 kb CpG island (20–26). Regulation of *GR* transcription has been extensively studied. At least 29 transcription factor binding sites have been experimentally confirmed, controlling first exon usage (24). Additionally, the CpG island promoters were shown to be susceptible to methylation, linking expression levels to the environment, fine-tuning *GR* levels (24–27).

By adapting the classical RNA ligase-mediated rapid amplification of 5′ cDNA ends (5′-RACE) to Next-Generation Sequencing (NGS) we were able to study the variability and complexity of the *GR* transcription initiation process in greater depth, identifying transcriptional initiation loci that themselves contain many, often adjacent, unique TSSs. The experimental protocol was designed to exclude any other potential interpretation of the NGS results and to minimise any potential ligation bias. The RNA oligo ligation strategy, employing TAP and CIP treatments, labelled only mature, undegraded mRNAs. By using a common RNA or DNA oligo, potential ligation bias between the sequences was reduced. On top of the experimental precautions, a 0.1% cut-off was introduced to define genuine TSSs, ensuring that errors introduced during the sample preparations are minimal and below our cut-off. The simple mono-exonic gene *ADRB2R* was used as a control. *ADRB2R* showed little transcriptional variability. Our data expands the unique literature TSS to one unique locus consisting of four adjacent TSSs. The multi-exonic *GR* gene on the other hand targeted a total of 358 TSSs throughout 38 loci that were cell line and stimuli specific. This microvariability around individual loci was negatively associated with translational efficiency and controlled the relative abundance of *GR* translational isoforms. Although this combination of techniques was initially intend to investigate the particular case of the *GR*, we suggest that our observations can be extended to other genes.

## MATERIALS AND METHODS

### Cell culture and RNA extraction

Three cell lines, DAUDI, MCF-7 and A549, were cultured as previously described (25,28,29). All culture media were from Lonza (Verviers, Belgium). T cells were isolated from PBMC's by Ficoll-isopaque (GE Healthcare Life Sciences, Amersham, UK) gradient centrifugation and by positive magnetic selection (Miltenyi midiMacs, Miltenyi Biotech GmbH, Cologne, Germany) (21).

Total RNA was extracted from unstimulated DAUDI, MCF-7 and A549 cells, following routine passage using the RNeasy Mini Kit (QIAGEN, Venlo, Netherlands) according to the manufacturer's instructions. 1.5 × 10^7^ DAUDI cells were seeded in a 75 cm^3^ flask. When they reached 70% confluence, they were stimulated with Interferon-γ (IFN-γ; 6 h, 5 ng/ml), Dexamethasone (Dex; 6 h, 100 nM) or 5-AZA-2′-deoxycytidine (AZA; 72 h, 10 μM) (Sigma-Aldrich, Diegem, Belgium). Cells were detached from the culture support using trypsin–EDTA (Lonza) and pelleted (5 min, 1671.6 × g). Subsequently, total RNA was isolated using the RNeasy Mini Kit (QIAGEN). RNA integrity was assessed using the Eukaryote Total RNA Nano assay with a RNA 6000 Nano chip on the Aligent 2100 Bioanalyzer (Aligent Technologies, Diegem, Belgium). The RNA quality assessment was based on the RNA integrity number (RIN). Only samples with a RIN value of >7 were used for further experiments.

### RACE-PCR

To label exclusively the 5′ nt of the mRNA, an RNA oligo was ligated in place of the mature mRNA-specific m^7^G cap structure, as previously described using the reagents from Invitrogen (Life Technologies, Paisley, UK) (30–32). Briefly, RNA was treated with calf intestinal phosphatase (CIP) to remove all active 5′ mono-phosphates from truncated or otherwise degraded mRNA as well as other RNA's, rendering those sequences unavailable for ligation and leaving only intact capped mRNA unaffected. Subsequently, tobacco acid pyrophosphatase (TAP) was used to remove the 5′ cap structure leaving a unique active 5′ phosphate on mature mRNA by hydrolysing the pyrophosphate bonds on the m^7^G cap triphosphate bridge. An RNA oligo (5′-CGACUGGAGCACGAGGACACUGACAUGGACUGAAGGAGUAGAAA-3′) was ligated to the unique active 5′ phosphate using a T4 RNA ligase in a 10 μl reaction containing 2 ng dephosphorylated, decapped RNA and 0.25 μg RNA Oligo, 10X Ligase Buffer, 10 mM ATP, 40 U/μl RNaseOut and 5 U/μl T4 RNA ligase. First-strand cDNA was obtained by reverse transcription of the ligated mRNA using a *GR*-specific primer in exon 2 (5′-CAGTGGATGCTGAACTCTTGG-3′, Eurogentec, Seraing, Belgium) or dN6 random hexamer primers (Invitrogen, Life Technologies), for the control gene (*ADRB2R*). Two rounds of PCR amplification were performed with forward primers located within the RNA oligo and reverse primers in exon 2 of the *GR* (Supplementary Data Table S1). The control gene was similarly amplified using the same RNA oligo specific forward primers and two *ADRB2R* specific reverse primers (Supplementary Data Table S1). The reverse primers were located respectively 29 bp downstream and 96 bp upstream of the ATG translation start codon. Amplification was performed in 25 μl reactions containing 20 mM Tris–HCl, 50 mM KCl, 2 mM MgCl_2_, 200 μM dNTPs, primers (Eurogentec), 1× SYBR green and 1.5 U Platinum Taq Polymerase (Life Technologies). Thermal cycling (CFX96, BioRad, Hercules, CA, USA) conditions were 95°C, 2 min; 45 cycles of 95°C 20 s, Ta 20 s, 72°C 90 s; and a final elongation step at 72°C for 10 min. Nested PCRs were performed using a 1:100 dilution of the first round PCR product as a template. Prior to NGS library preparation, the PCR products were purified using Agent AmPure XP Beads (Analis, Suarlée, Belgium) and quantified with the Quant-iT picogreen dsDNA Assay Kit (Life Technologies) according to the manufacturers’ instructions.

### Next-Generation Sequencing

The NGS libraries were prepared using the Ion Xpress Plus Fragment Library Kit (Rev. A, Life Technologies) according to the manufacturer's instructions for 100 ng gDNA. Briefly, purified PCR products were sheared (Ion Shear Plus Kit, Life Technologies) and Ion Xpress Barcode Adapters (Life Technologies) were ligated to the resulting DNA strands. Using 2% agarose gels (E-gel System, Life Technologies), DNA fragments of 200–350nt length were selected for further amplification. The DNA concentration was estimated with the Agilent High Sensitivity DNA chip on the Agilent Bioanalyzer (Agilent Technologies) and equimolar quantities of each library were pooled (*GR* gene: 4 libraries; *ADRB2R* gene: 12 libraries).

Template preparation was carried out using the Ion OneTouch 200 System Template Kit v2 protocol (Rev. 4, Life Technologies) recommended by the manufacturer. Briefly, a diluted library pool was added to the emulsion mix for DNA clonal amplification on the Ion OneTouch instrument, followed by an enrichment of the template-positive Ion Sphere Particles (ISP) on the Ion OneTouch ES instrument (Life Technologies). To assess the quality and calculate the appropriate library dilution, both unenriched and enriched ISP samples were qualified and quantified performing the Qubit™ dsDNA HS Assay Kits (Life Technologies) using a Qubit 2.0 Fluorometer (Life Technologies) according to the manufacturer's protocol.

Ion Torrent PGM runs were performed using the Ion PGM 200 Sequencing Kit (PN4474246 Rev. D, Life Technologies) on Ion 314 and 316 Chips (Life Technologies), as simplex and multiplexed runs, with the standard Torrent Suite parameters (Supplementary Data Figure S2A).

### Bio-informatics and statistical analyses

NGS sequencing reads were processed using the default Torrent Suite settings. Sequences containing at least the last 8 nt of the 5′ cap oligo after Multiplex Identifier (MID) sorting were retained as oligo-labelled TSSs for further analysis, all non-labelled sequences were discarded. This 5′ cap oligo sequence was subsequently trimmed and the reads were mapped against the genomic reference for the *GR* gene (Chr5, hg19, 142 657 496 to 142 850 254) with TopHat software (33,34) (v2.0.3), using default settings. A python script retrieved the TSS for each aligned read. Oligo-labelled TSS reads were also analysed manually with Geneious software (Biomatters, v5.5.6). Throughout this study, *GR* TSSs are annotated with respect to the ATG (+1) translation initiation codon and the *ADRB2R* TSSs with respect to the first nt in the mRNA sequence (NM_000024.5).

TSS count data were normalised using the ‘Trimmed Mean of the *M*-values’ (TMM) technique in R (R Core Team 2014). R: A language and environment for statistical computing. R Foundation for Statistical Computing, Vienna, Austria. URL http://www.R-project.org/ v3.0.2) using the Bioconductor package NOISeq (35) (v2.6.0). To remove background noise, all TSSs corresponding to a sequence frequency <0.1% in replicate runs were removed. Data were visualized in Bioconductor packages limma (36) (v3.18.13) and affycoretools (MacDonald, J.W. (2008). affycoretools: Functions useful for those doing repetitive analyses with Affymetrix GeneChips. R package version 1.34.0). Differential expression analysis was performed with Bioconductor package NOISeq (35) (v2.6.0). Differentially expressed TSSs were hierarchically clustered and visualised using CRAN-packages cluster (Maechler, M., Rousseeuw, P., Struyf, A., Hubert, M., Hornik, K. (2014) cluster: Cluster Analysis Basics and Extensions. R package version 1.15.2) and pheatmap (Raivo, K. (2013). pheatmap: Pretty Heatmaps. R package version 0.7.7).

The evolutionary conservation was visualised with the UCSC browser (https://genome-euro.ucsc.edu). Differentially expressed TSS frequencies were plotted against the PHAST phyloP conservation score from the publically available 100 vertebrate genome alignment (37). *In silico* phylogenetic footprints (ISPF) were obtained from a previous report (38).

### Translational efficiency of transcriptional micro-variants

The previously reported full-length exons and the CMV promoter were cloned into the synthetic firefly luciferase pGL 4.10 vector (Supplementary Data Figure S3) (Promega, Leiden, Netherlands) (11). Shorter 5' microvariants of the exons, identified by NGS, were synthesized and also inserted into pGL4.10 (GeneCust, Dudelange, Luxembourg) (Supplementary Data Table S2).

Twenty-four hours prior to transfection A549 cells were seeded into 24-well plates (4 × 10^4^ cells/well). Cells were transfected with 750 ng of 5′ UTR constructs, using 0.5 μl of PLUS Reagent and 2.0 μl of Lipofectamine LTX (Life Technologies) according to the manufacturer's instructions. The *Renilla* luciferase plasmid pGL 4.73 was used as control vector and cells were transfected using a 10:1 ratio of the two plasmids.

The firefly and *Renilla* luciferase activity were measured using the Dual-Glo Luciferase assay system (Promega) according to the manufacturer's protocol. The luminescent signal was read with Infinite M200 plate reader (TECAN, Männedorf, Switzerland). The experiments were performed in biological triplicates. Within exon variants, the luminescent signals were subjected to a pairwise multiple comparison using Kruskal–Wallis One Way Analysis of Variance on Ranks with a Tukey post-hoc correction, reporting q-values for a type I error level of 0.05 per comparison.

### RNA structure prediction

The free energy released on RNA folding (ΔG) and the resultant secondary structure of the complete *GR* transcripts and of the individual 5′UTRs were calculated using the online RNAfold algorithm (http://rna.tbi.univie.ac.at/cgi-bin/RNAfold.cgi) (39). Default settings were used for all predictions.

### Minigene design & plasmid construction

The plasmids were constructed as previously described (11). Briefly, all constructs contained a first exon variant 1A3, 1B or 1C (Supplementary Data, Table S2), followed by exons 2 till 8 (NM_000176.2; nt 480–2673) and the genomic sequence of exon 9α and the corresponding introns (NCBI36/hg18 release March 2006, chr5: 142 637 665–142 642 326). A total of 10 different length constructs were prepared. All inserts were synthesized (Genecust, Dudelange, Luxembourg) and subsequently cloned into pcDNA3.1 (–) (Life Technologies, Merelbeke, Belgium).

### Western blot

GR protein isoform quantification was performed as previously described (11). Briefly, A549 cells were transfected with 500ng DNA using Lipofectamine LTX (Life Technologies) 24 h post-seeding. Total proteins were extracted forty-eight hours post transfection, separated on 4–12% Bis–Tris ZOOM™ gels (Life Technologies) and immunoblotted with primary rabbit-α-GR antibody (P20 clone, epitope within aa 720–769 of the hGRα; Santa Cruz Biotechnologies, Heidelberg, Germany) and Cy5-labeled secondary antibody (GE Healthcare). After washing, mouse anti-β-actin (anti-β-actin, Santa Cruz Biotechnologies) probing and secondary goat-anti-mouse Cy3 antibody (GE Healthcare) incubation, the immunoreactive bands on the membrane were read using the Typhoon 9400 imager (GE Healthcare) at excitation wavelength 633nm and 532nm for Cy5 and Cy3 respectively (PMT = 480V; scanning resolution = 50 μm). Band intensities were quantified using ImageJ (NIH, Bethesda, MD, USA) and normalized according to β-actin. Variance was determined with One-Way or Two-Way ANOVAs (Sigmaplot 12.3), pairwise comparisons were performed with the Student–Newman–Keuls's test and *P* < 0.05 were considered significant.

## RESULTS

### 5′-RACE library sequencing

#### Library preparation and sequencing quality

To investigate the variability in TSS usage of the *ADRB2R* and *GR* gene, RNA ligase mediated rapid amplification of 5′ cDNA ends (5′-RACE) was adapted to massively parallel sequencing as outlined in Figure 1C and D. Ion Sphere loading densities (ISP) of ∼68% were observed, corresponding to >7.4 × 10^5^ reads and >4.4 × 10^6^ reads for *ABRB2R* libraries on 314 and 316 chips respectively. Removing polyclonal reads reduced the numbers of reads to 3.6 × 10^5^ and 3.2 × 10^6^ reads, respectively. *GR* libraries had ISP densities of ∼60% with >3.7 × 10^6^ reads per 316 chip. These were reduced to 2.3 × 10^6^ after removal of ∼23–24% polyclonal reads. Sequence quality, assessed by PHRED values, for both genes was unaffected by the 5′-RACE library preparation. PHRED values were >25 and fell below the acceptable quality threshold of a PHRED score of 20 only after 200 nt, as expected from the Ion PGM 200 Sequencing Kit. In all multiplex *ADRB2R* and *GR* sequencing data sets, reads were equally distributed over the different MIDs. To identify m^7^G-capped TSSs, only 5′ oligo-labelled sequences were analysed. Oligo selection and trimming produced data sets of 170 000 to 800 000 reads and 193 727 to 378 859 reads for the *GR* and *ADRB2R* gene that were retained as labelled TSSs. Because of the shearing step in the library preparation, on average only 40% of these reads (89 000 to 350 000 reads) contained a labelled 5′ TSS. However, 97% (186 608 to 366 431) of these 5′ TSS reads were successfully mapped against the *GR* or the *ADRB2R* gene region. Overall, the aligned reads corresponded to respectively 22–65% and 69–95% of the initial reads per sample. All raw and aligned sequencing data are available on the European Nucleotide Archive (ENA) of the EMBL-EBI under accession number PRJEB9064.

#### GC content does not influence sequencing

The *ADRB2R* and the *GR* differ significantly in their 5′ G+C content. This resulted in a somewhat lower loading efficiency (range 52–68%) for the *GR* with the higher G+C content than the *ADRB2R* (range 59–74%) with the lower G+C content. The sequence quality of both genes was similar, with only slightly higher PHRED values for *ADRB2R* (>28) than for *GR* (>25). Thus, the difference in CG content did not seem to affect the sequencing quality.

#### Sequencing artefacts do not perturb TSS identification

Analysis of the NGS reads revealed no substitutions in either the TSSs of the *ADRB2R* gene (0.187 × 10^6^ sequences/21.9 × 10^6^ total nt sequenced) or the *GR* gene (0.258 × 10^6^ sequences/50.2 × 10^6^ total nt sequenced). As expected for Ion Torrent sequencing, however, the rate of insertions and deletions (indels) was high. We observed a 6% indel rate (0.05 insertions per total nt sequenced and 0.005 deletions per total nt sequenced) for *ADRB2R* and 2.20% for *GR* (0.007 insertions per total nt sequenced and 0.004 deletions per total nt sequenced). Importantly, none of these indels were observed in the TSS region, i.e. in the 3′ end of the RNA oligo or in the nt immediately downstream of the TSS. The TSSs were also checked for homopolymers. Only one of the 21 most important differentially expressed TSSs identified in the *GR* was part of a two nt homopolymer. The *ADRB2R* gene had no homopolymer in its first exon region. Thus neither substitution, nor indels or homopolymers compromised TSS identification.

#### The alignment method does not affect the TSS identification

**Figure 2.**
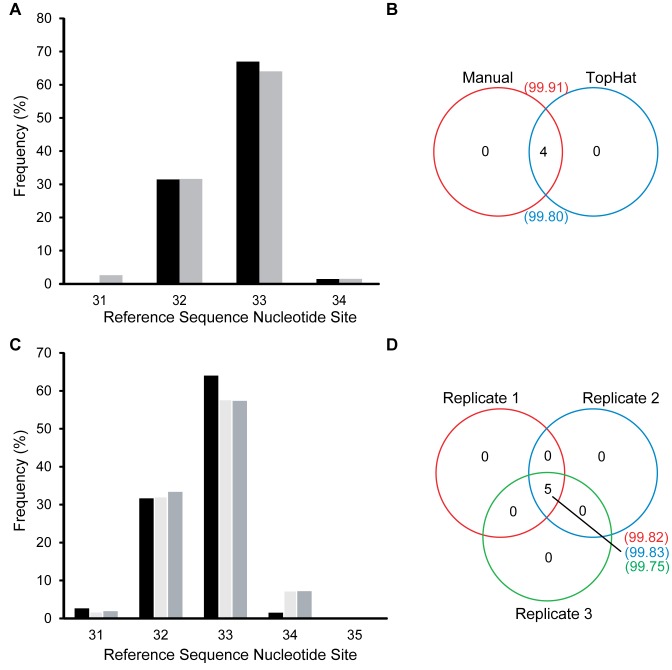
The mono-exonic *ADRB2R* shows transcriptional microvariability around one locus. (**A**) Frequency of reads aligned to individual TSSs using TopHat (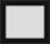) and a manual approach (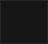). (**B**) The number of TSSs shared between data sets aligned using TopHat and the manual approach. Numbers in brackets are the frequency (%). (**D**) Bar plot representing the frequency (%) of reads aligned per TSS for three biological replicates. Replicate 1 (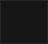); Replicate 2 (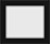); Replicate 3 (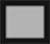) (**E**) The number of TSSs shared between the biological replicates and the frequency of reads (%) per NGS run corresponding to these TSSs.

Since the TopHat alignment algorithm is relatively strict, sequences with sequencing errors and indels may have been excluded from the aligned data sets. Therefore, the analysis was repeated using Genious with less restrictive parameters and the alignment of each sequence was manually verified. The Genious and TopHat alignments, both mapped similar read numbers (309 739 sequences, 99.76% *versus* 308 341 sequences, 99.31%) against the *ABRB2R* reference sequence and both approaches identified the same TSSs with virtually identical frequencies (Figure 2A and B). Both methods selected 7 TSSs: two thirds (64–67%) of the reads started at nt position 33, irrespective of the alignment method, One third (range 31.40–31.64%) of the reads started at nt position 32. Read numbers starting at nt 31 and 34 accounted for less than 3%. Percentages of reads with TSSs at nt 30, 35 and 39 were <0.10%, and may represent the error rate in TSS identification. Therefore, a cut-off of <0.10% was applied for all subsequent runs to validate TSSs. As there was virtually no difference in mapping results between the two methods, TopHat was used in all subsequent analyses.

#### Sequencing depth requirements

In addition, to determine the read depth necessary to identify valid TSSs, technical triplicates of the *GR* in DAUDI cells were performed on 314 and 316 chips, either in simplex or multiplex format, with similar loading and mapping values (Supplementary Data Figure S2A). Despite generating different read numbers for the different NGS conditions, the TSSs pattern between the technical replicates was conserved (Supplementary Data, Figure S2A and S2B). On average 97% of the sequences per replicate run target one of the 103 shared TSSs. Fewer than 4 TSSs were common to only two of the replicates, and none had unique TSSs (Supplementary Data, Figure S2B). These data suggest that for the 3 kb variable *GR* TSS region, a total number of oligo-labelled reads equal to 50× the length of the variable region, is adequate to detect microvariable TSSs.

### The mono-exonic *ADRB2R* gene shows biological microvariability around one transcriptional locus

Analysis of a series of biological replicates revealed that 99.79% (±0.4) of the mRNA sequences used one of five consecutive nt (31–35) within the published sequence (Figure 2C). The relative TSS frequencies were essentially identical between replicates (Figure 2D). TSS 35 usage was minimal (range 0.02–0.14%) and was therefore excluded as a valid TSS. The TSSs 30 and 39, mentioned in the previous section, were excluded as valid TSSs, after the application of the 0.10% cut-off. We conclude that the *ADRB2R* microvariability is limited to 4 nt around a single TSS locus.

### The *GR* TSSs are highly variable

**Figure 3.**
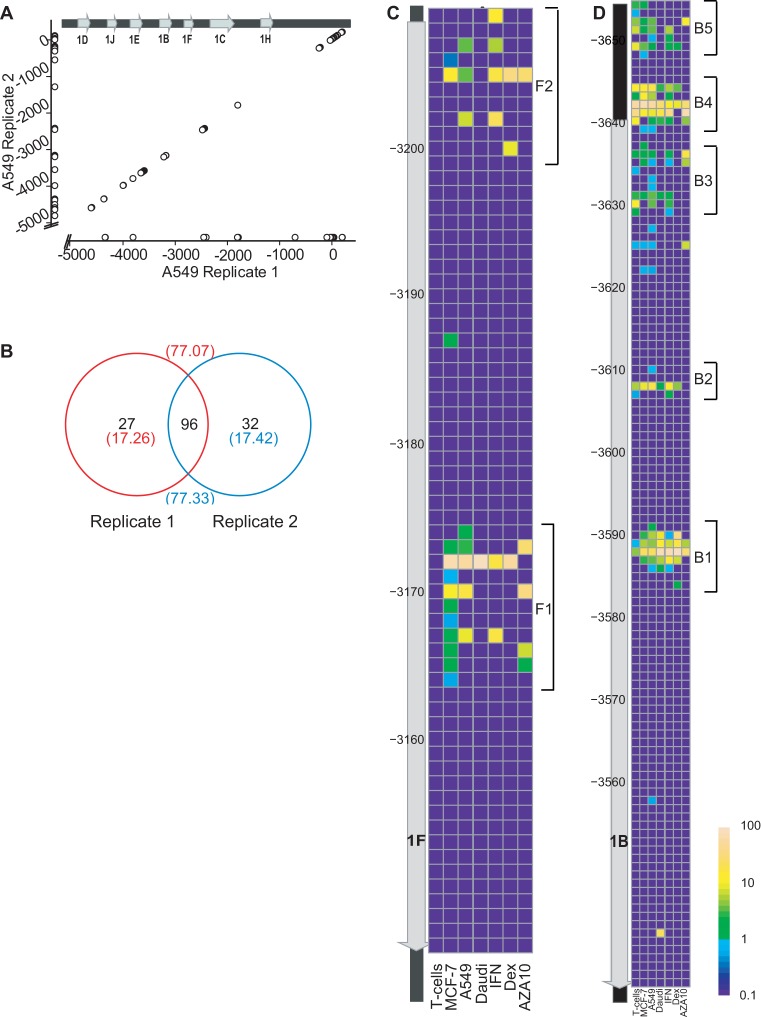
Microvariable TSS distribution throughout *NR3C1*. (**A**) The proximal *NR3C1* CpG island TSSs used in two A549 biological replicates are plotted showing common and unique TSSs. The *NR3C1* TSSs on both axes are annotated with respect to the ATG (±1) translation initiation codon in exon 2. Data points adjacent to the x- and y-axes are unique to the respective replicates. (**B**) The number of TSSs shared between A549 biological replicates (numbers in parentheses are the% of total oligo-labelled 5′ TSSs). (**C**) Detailed TSS usage pattern for the four cell lines and three treatment condition for exon 1F. (**D**) Detailed TSS usage pattern for the four cell lines and three treatment condition for exon 1B. Published exon 1F and 1B locations (21) are shown as a gray arrow, on the left of the heatmap. The TSS usage is expressed as the log value of the percentage of TSS expression for specific exon, by colour [0.01% (blue) to 100% (yellow)] of the heatmap.

The *GR* 5′ UTR of different cell lines and after different cell treatments were sequenced using the same protocol as for *ADRB2R*. Sequencing libraries were made from templates covering a region from the oligo-labelled TSSs to the start of the common exon 2. The oligo-labelled TSS reads were successfully aligned by TopHat to the *GR*. Each of the nine *GR* first exons showed a remarkable TSS variability, but all microvariants used the previously published 3′ splice donor sites (Figure 1B). For example, in the biological replicates of A549 cells, we identified 123–128 TSSs, of which 96 were shared between replicates. The 96 shared TSSs accounted for ∼77% of the reads per run (Figure 3A and B). About ∼17% of the reads used 27–32 unique TSSs, accounting each for 0.11–4.39% of the total reads per TSS. The remaining ∼6% of the reads correspond to TSSs below the 0.10% cut-off and represent the intrinsic identification error rate. The TSSs were distributed in multiple loci per exon throughout the CpG island (Figure 3A). Each cluster, or locus, consisted of a series of adjacent TSSs, that we term microvariability. For example, exon 1F was previously reported to be 62 nt long, starting at −3208 and ending −3146 (21). We observed a series of shorter 1F exons, with two clusters of transcriptional loci around TSSs −3205 ±4bp and −3170 ±4bp all sharing the −3536 splice donor site (Figure 3C). Exon 1B, with a length of 104 nt (−3640 to −3536), had 24 shorter forms all sharing the common splice donor site at −3146 (Figure 3D) (40) and 11 TSSs immediately upstream of the TSS reported in the literature(21,23). Similar trends including multiple microvariable transcriptional loci per exon were also observed for the regions corresponding to the other CpG island exons (1C, 1D, 1E, 1H and 1J) and the distal exons (1A and 1I) (Supplementary Data Figures S4–S7). Some of the TSSs observed here, were located within a region immediately upstream of the ATG translation initiation codon in exon 2, and interestingly even downstream of the ATG, but still within exon 2. In general, our alternative TSSs were shorter than previously reported (21,23), although, for instance locus B4 and B5 were upstream of the previously identified TSS (Figure 3D). Both the TSSs used and their variability, were reproducibly cell line dependent. This pattern of reproducible microvariability around multiple transcriptional loci between biological replicates was also observed for MCF-7 and T cells (Supplementary Data Figures S8 and S9).

### TSS expression profiles are cell line specific

**Figure 4.**
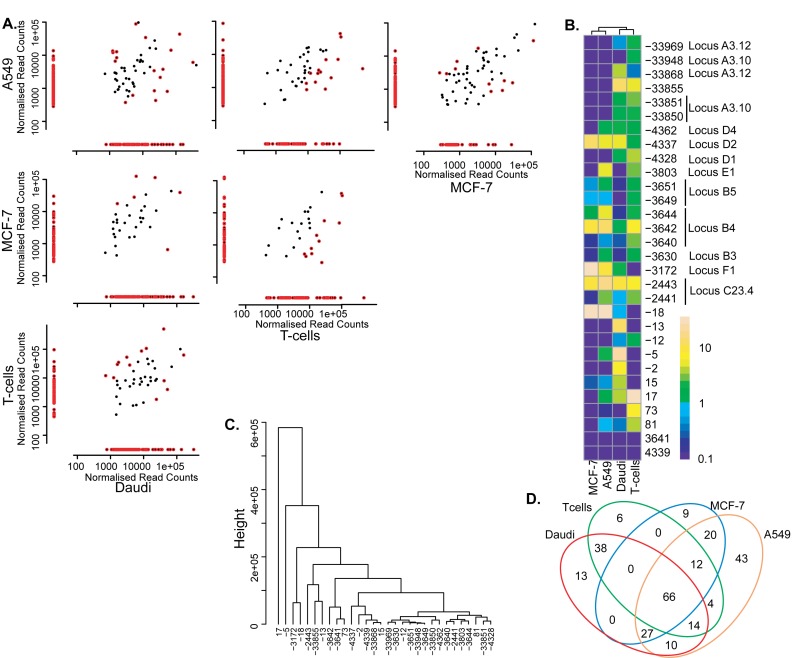
Differential expression of TSSs between multiple cell lines. (**A**) Pairwise comparisons of normalised read counts for all cell lines. The coloured dots (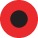) represent the significantly differentially expressed TSSs between two cell lines, the black dots (•) represent TSSs that are not differentially expressed between two cell lines. Significance was considered for an adjusted *P*-value of 0.05 after performing the NOISeq proportion test (35). (**B**) Expression of the 30 most discriminatory TSSs between the different cell lines, as a percentage of the total oligo-labelled TSSs. (**C**) Hierarchical clustering of the 30 most discriminatory differentially expressed TSSs. (**D**) The number of unique and common differentially expressed TSSs for the four cell lines.

In total we observed 262 discrete TSSs throughout the proximal and distal promoter for the four cell lines aggregated (A549, MCF-7, T-cells and DAUDI) (Figure 4B and D). Sixty six of these were common to all cell lines and other were shared with at least one other cell line. Each cell line had a small population of unique TSSs (range 6–43 TSSs). Pairwise analysis of the four cell lines, revealed between 134 and 196 TSSs that were differentially expressed per comparison (Figure 4A). Some of these differential TSSs were unique to one cell line, while others were used in several cell lines at similar or different levels. For example, 125 TSSs were shared by both MCF-7 and A549 cells (Figure 4A and D), while nine were uniquely expressed in MCF-7 and were not seen in any other cell lines. 71 TSSs were solely expressed in A549 and not in MCF-7. 28 of these were also found in other cell lines, leaving 43 TSSs unique to A549 cells. Hierarchical clustering identified the 30 TSSs that most strongly discriminated between the cell lines (Figure 4B and C). Many of these could be related to transcriptional loci observed for exon 1B and 1F (Figure 3C and D). When the read frequencies were taken into account, a smaller group of eight TSSs emerged, which clearly discriminated between the different cell lines (A549, MCF-7, T-cells and DAUDI) (Figure 4B). The above eight TSSs were also the most conspicuous within the dendrogram (Figure 4C). The TSSs 17 and −18 were T cells specific, TSSs −5, −13, −3642 (locus B4) and −33 855 were DAUDI cell specific and TSSs −3172 (locus F1) and −2443 (locus C23.4) differentiated best between MCF-7 and the A549 cell line respectively. Except TSSs 17 and −33 855, these characteristic TSSs were located within the proximal promoter. TSSs −5, −13, −18 and 17 were located within exon 2. The first three were situated upstream and the latter downstream of the ATG translation initiation codon in exon 2. The TSSs −2443, −3172 and −3642 correspond to exons 1C, 1F and 1B in the proximal promoter region. TSS −33 855 corresponds to exon 1A in the distal promoter region. The remaining differentially expressed TSSs also differed between cell lines, yet, in a much less obvious way.

### DAUDI cells have highly variable biological replicates

In contrast to other cell lines, the TSS selection patterns in DAUDI cells were less consistent, even in multiple biological replicates (Supplementary Data Figure S10). The DAUDI cell line, a Human Birkitt's lymphoma cell line that reliably expresses the distant 1A exon, selected TSSs in both the distal and proximal promoter regions.

To assess the importance of read depth and determine potential sources of biological variability from technical variability, technical triplicates of the *GR* in DAUDI cells were sequenced on respectively 314, 316 and multiplexed 316 chips with different MIDs. Resulting in respectively 61 807, 2 338 506 and 639 608 reads per run. After applying the 0.10% cut-off, 103 TSS, which corresponded to 97.14–98.09% of all reads, were shared between all replicates (Supplementary Data Figure S2B). As expected, differential expression analysis did not identify any discriminating or run-specific TSS expression profile. Hereby, indicating that the error induced by differences in MIDs, adaptors, PCR cycles and Ion Torrent chips was only minimal and confirmed the biological origin of the variability in the Daudi cells.

### Environmental conditions influence the TSS pattern

**Figure 5.**
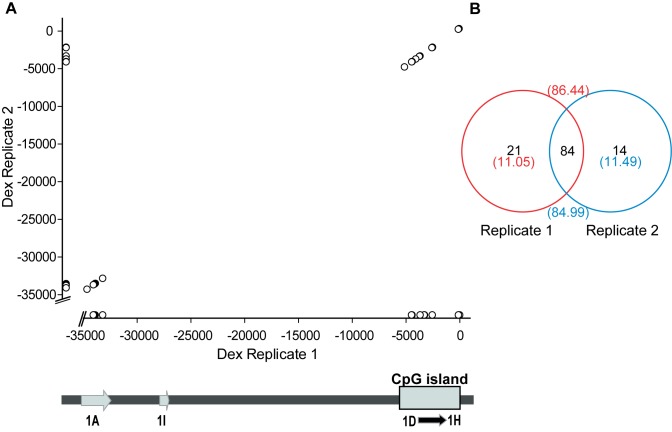
Microvariable TSS distribution throughout the *NR3C1* under different treatment conditions. (**A**) Reproducibility of TSS identification in DAUDI cells after dexamethasone stimulation. The region covered corresponds to both the distal and proximal promoter regions of *NR3C1*. (**B**) The number of TSSs shared between the two dexamethasone stimulation replicates (numbers in parentheses are the% of total oligo-labelled 5′ TSSs).

To examine the effect of transcriptional stimuli on TSS microvariability, the transcriptionally variable DAUDI cells were exposed to IFN-γ, Dex or AZA. These treatments markedly reduced the TSS variability observed in unstimulated DAUDI cells. TSS selection patterns became reproducible between biological replicates, but differed between treatments. When exposed to Dex, DAUDI cell replicates targeted 98–105 TSSs, of which 84 were shared between replicates. The 84 shared TSSs accounted for ∼85% of the reads per run (Figure 5A and B). As for the experiments above with stable unstimulated MCF-7, A549 and T cells, each run also selected a small number of unique TSSs (range 14–21), but these correspond to not >11.49% of the reads and less than 3% of the total reads per TSS. The remaining ∼4% of the reads were below the 0.1% cut-off and represent the error rate intrinsic to our 5′ labeling technique. Although the majority of the TSSs were located within the proximal promoter region, a smaller number of TSSs were found in the distal *GR* promoter region (Figure 7A). Similar results were observed for DAUDI cells exposed to IFN-γ or AZA (Supplementary Data Figures S11 and S12).

**Figure 6.**
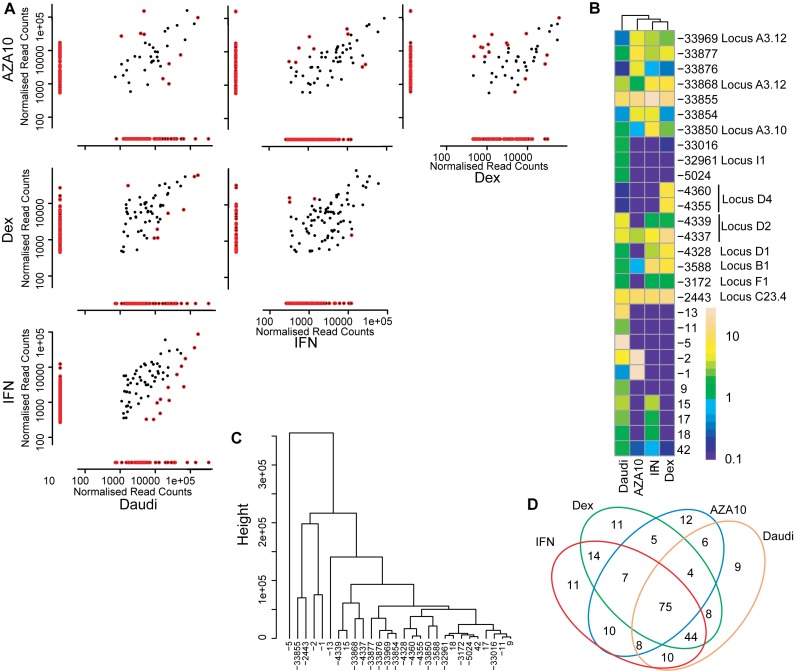
Differential expression of *NR3C1* TSSs between untreated, dexamethasone, Interferon-γ and 5-AZA-2′-deoxcytidine exposed DAUDI cells. (**A**) Pairwise comparisons of normalised read counts for all cell lines. Only valid TSSs with a frequency above 0.1% of the total reads were included. The coloured dots (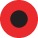) represent the significantly differentially expressed TSSs between cell treatment conditions, the black dots (•) represent TSSs that are not non-differentially expressed between treatments. Significance was considered for an adjusted *p*-value of 0.05 after performing the NOISeq proportion test (35). (**B**) Heatmap of the 30 most discriminatory TSSs between the different treatment conditions, expressed on a logarithmic scale. (**C**) Hierarchical clustering of the 30 most discriminatory differentially expressed TSSs. (**D**) The number of unique and common differentially expressed TSSs between the different treatment conditions untreated, dexamethasone, Interferon-γ and 5-AZA-2′-deoxcytidine.

In total, we observed 234 discrete TSSs throughout the proximal and distal promoter region for the four treatments aggregated (Figure 6B and D). Seventy-five of these were common to all treatments and other were shared with at least one other treatment. Each treatment had a small population of unique TSSs (range 9–12 TSSs). Pairwise analysis of the different treatments, revealed between 127 and 179 TSSs that were differentially expressed per comparison (Figure 6A). Similar to the comparison between cell lines, some of these differential TSSs were unique to one treatment, while others were used in several treatments at similar or different levels. For example, 140 TSSs were shared by both Dex and IFN-γ stimulations (Figure 6A and D), while 28 and 39 TSSs were solely expressed after Dex or IFN-γ treatment respectively. 17 of the 28 TSSs induced by Dex were also found in other treatments, leaving 11 TSSs unique to Dex. Similarly, 27 of the 39 TSSs induced by IFN-γ were also associated with other treatments, leaving 11 TSSs unique to IFN-γ. Hierarchical clustering identified 28 TSSs that most strongly discriminated between treatments (Figure 6B and C). Many could be related to transcriptional loci observed in exon 1B and 1F (Figure 3C and D). When the read frequencies were taken into account, a small group of six TSSs emerged, which clearly discriminated between the different treatments (Figure 6B). The above six TSSs were also the most conspicuous within the dendrogram (Figure 6C). The TSSs −5 and −13 were specific to untreated DAUDI cells, TSSs −1 and −2 were DAUDI cells + AZA specific and TSSs -2443 (locus C23.4) and −33 855 differentiated best between IFN-γ and Dex treatments respectively. TSSs −1, −2, −5 and −13 were situated in exon 2, upstream of its ATG translation initiation codon. The TSS −2443 (locus C23.4) corresponds to exon 1C in the proximal promoter region and TSS −33 855 corresponds to exon 1A3 in the distal promoter region. The remaining differentially expressed TSSs also differentiated between different stimulations, yet, in a much less obvious way. As in our comparison of resting cell lines, we observed transcriptional microvariability around the same loci for the DAUDI cells exposed to either IFN-γ, AZA or Dex. Although, transcription microvariability was observed to occur in the same loci as unstimulated cells, the differential expression pattern, i.e. the pattern of TSSs within each locus, was treatment specific.

### Translational efficiency assay

**Figure 7.**
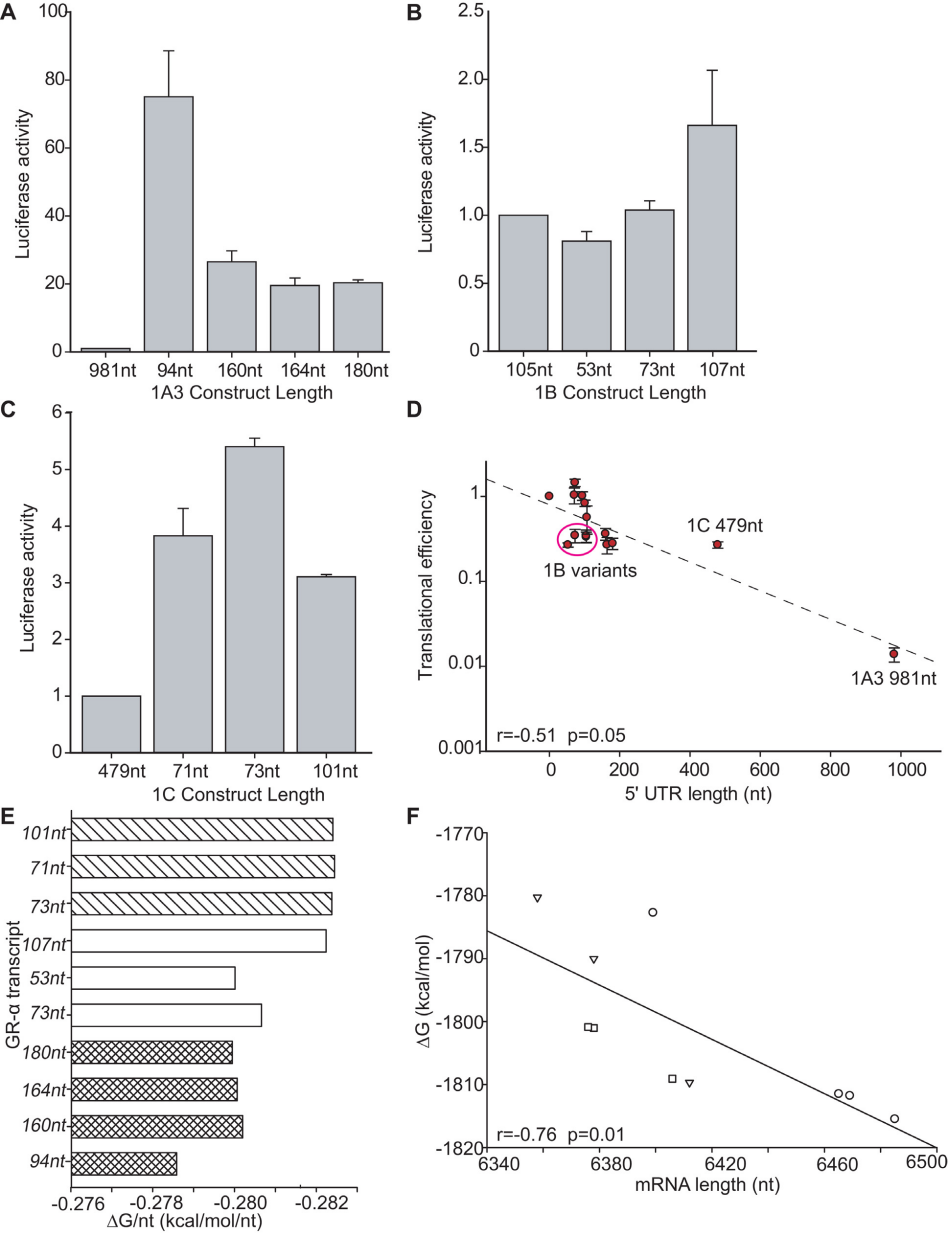
Translational efficiency and mRNA stability for *NR3C1* exon 1A, 1B and 1C. (**A**) The luciferase activity of three replicates per 1A3 construct for different construct lengths. The first construct being the original. The luciferase state of the different constructs was normalised to the luciferase activity of the original full length exons (11). (**B**) The luciferase activity of three replicates per 1B construct for different construct lengths. The first construct being the original. The luciferase state of the different constructs was normalised to the luciferase activity of the original full length exons (11). (**C**) The luciferase activity of three replicates per 1C construct for different construct lengths. The first construct being the original. The luciferase state of the different constructs was normalised to the luciferase activity of the original full length exons (11). (**D**) Translational efficiency values from luciferase are plotted in function of their 5′ UTR length expressed in base pairs (bp), presenting a linear decrease in efficiency as the length increases. Exon 1B variant with a lower translation efficiency/nt are circled, and the marked 1C and 1A3 datapoints are the full length literature sequences (20,40). (**E**) The free energy of folding (ΔG/nt) for 1A3 microvariable sequences calculated per nucleotide (1A3: 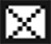; 1B 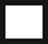; 1C 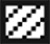) constructs. (**F**) The mRNA length in function of the free folding energy of the mRNA (Δ*G*) (1A3: 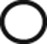; 1B: 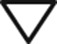; 1C: 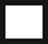). Data in panels A–D are mean ± SD.

To investigate the functional consequence of *GR* microvariability, we constructed a series of 14 plasmids. Each plasmid contained a first exon variant from of 1A3, 1B or 1C, immediately upstream of a luciferase coding sequence. The first exon microvariants were either (i) full length first exon sequences as reported in the literature (21,23), (ii) a sequence starting from a TSS observed within a few nt of the literature start site until the end (e.g. 1B 105 bp and 107 bp), (iii) a sequence from TSSs mid first exon until the end (e.g. 1B 73 bp or 1C 71 bp and 73 bp) or (iv) a sequence starting from TSSs close to the 3′ end of the first exon to the end of the exon (e.g. 1A3 94 bp and 1B 53 bp). Luciferase assays were performed to evaluate the translational efficiency of the different 5′ UTRs (Figure 7D). The translation efficiency per exon decreased with the increase of construct's length. Translation efficiency of the 1B microvariability variants (highlighted in Figure 7D) was much lower than the 1A3 and 1C variants. In all cases, there was a considerable effect of the observed transcriptional microvariability on the efficiency of luciferase production (Figure 7A–C). The highest luciferase signal was observed for the 94 bp long exon 1A3 with a 75-fold increase (*q* = 4.65) compared to the full length first exon sequences as reported in the literature (21,23). Exon 1C (73bp) and exon 1B (107bp) showed the highest luciferase signal of any of the exon 1C and 1B constructs, with a 5-fold (*q* = 4.32) and a 2-fold increase (*q* = 2.40) respectively. Aside from the 1B constructs, that did not show any significant effect in luciferase activity, our observations were compatible with our prior report of shorter sequences having a higher activity (11). Accordingly, only small differences in activity were observed between transcripts of similar length, i.e. with neighbouring TSSs, e.g. 1C 71 bp versus 73 bp (*q* = 1.44) and 1A3 160 bp *versus* 164 bp (*q* = 1.807). The luciferase signal relative to the full length of the first exon was in general higher for the exons 1A3 transcriptional variants. The signals measured for exon 1C variants were only slightly higher compared to those of exon 1B variants. Although one could assess the previously published full length exons 1A3 and 1C, respectively 981nt and 479nt, as outliers (Figure 7D), removing them maintained the negative correlation and resulted in a steeper slope. Overall, translation efficiency increased as 5′ UTR length decreased, irrespective of the alternative first exon.

### *GR*-transcript secondary RNA structure is influenced by the 5′ UTR length

Secondary structures of full-length mature mRNA sequences of *GR*-α transcripts were modelled using the program RNAfold (39). The *GR*-α transcripts for exon 1A3, 1B and 1C all induce a similar folding. Overall there was a strong negative correlation between the total ΔG and the mRNA length irrespective of its location within the CpG island (*r* = −0.76) (Figure 7E). However, as would be expected based on the similarity of their sequences, microvariable sequences clearly cluster by exon. Within these populations the trend was even more pronounced (*r* = −0.99, *r* = −0.99, *r* = −0.99). The free energy per nucleotide (Δ*G*/nt), being a better indicator of the secondary mRNA structure stability, was anticipated to be similar between the 3 exon *GR* transcripts. The ΔG/nt values range from −0.2824 to −0.2786 for all three exon transcripts (Figure 7F). The ΔG/nt of *GR* 1A3 transcripts are slightly higher than those of the *GR* 1B and 1C transcripts, indicating that the 1A3 transcripts are probably slightly less stable than the 107nt long 1B exon construct or the 1C constructs. For *GR* 1C transcripts the ΔG/nt does not change with mRNA length, for the other exon microvariants, it is always the smallest mRNA variant that has the highest ΔG/nt. However all of these differences are minimal, therefore we would assume that the differences in stability are minimal.

### 5′UTRs influence the relative GR protein isoform distribution

**Figure 8.**
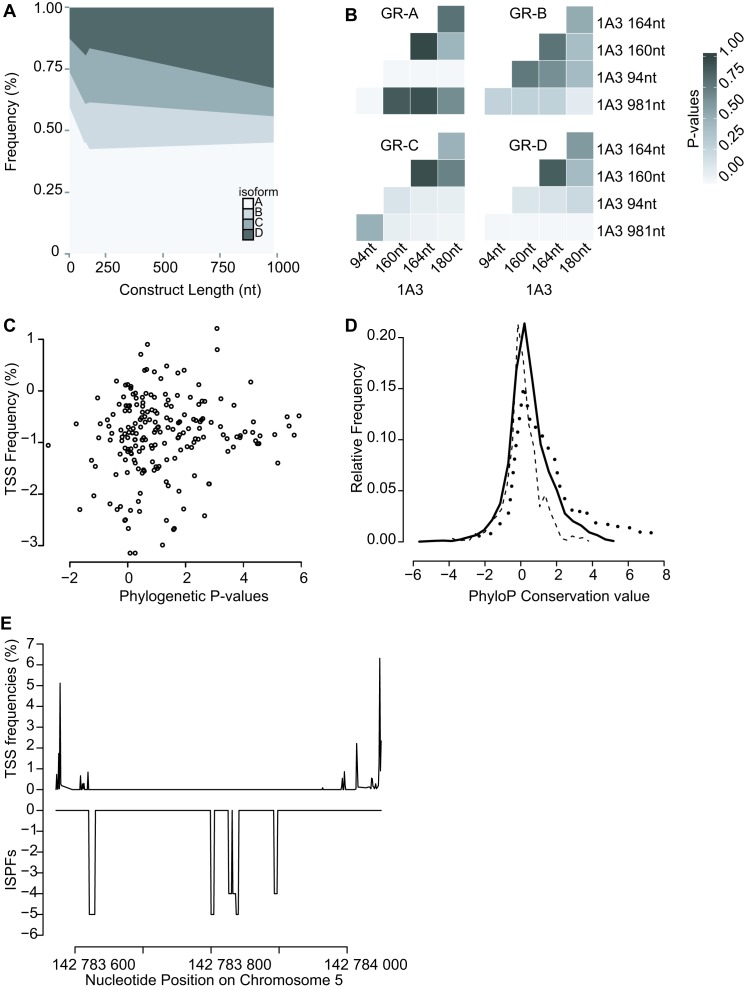
The microvariable TSSs influence the relative abundance of N-terminal protein issoforms and the evolutionary conservation of this region. (**A**) Areaplot for 1A3 microvariable constructs, indicating the shift of isoform levels according to the construct length. Two-Way ANOVA (DF = 12, *F* = 9.064, *P* < 0.01). (**B**) Pairwise comparison of relative protein levels. Student–Newman–Keuls *P*-values are expressed by grey scale [0.01 (white) to 1 (black)]. (**C**) The phylogenetic p-value of a specific nucleotide position is plotted in function of the TSS frequency measured at that specific location. (**D**) The relative frequency distribution for respectively the distal promoter (**—**), proximal promoter (– –) and exon 2 region (- - -) in function of the PhyloP conservation value. Negative values indicate higher evolution rates, positive values indicate higher conservation rates and zero equal a neutral position concerning evolution rates. (**E**) For exon 1F and the region before, the *in silico* phylogenetic footprinting sites (38) the TSS frequencies were plotted against one another.

To investigate the role of alternative 5′UTRs in translational start site selection, ten microvariable constructs covering the two constitutive (21) and the upstream distal first exon were made. The TSSs incorporated covered locus 142 814 191, 142 814 257, 142 814 261 and 142 814 277 for 1A3, 142 783 994, 142 784 014 and 142 784 048 for 1B and 142 782 847, 142 782 849 and 142 782 877 for 1C. Forty-eight hours post transfection all microvariable constructs showed significant differences in the distribution of N-terminal isoforms on western blots (Figure 8 and Supplementary Data Figure S13). The most abundant N-isoform for all constructs was GR-A (Figure 8A and Supplementary Data Figure S13). For 1A3 and 1C constructs, the GR-A isoform represented 50% of the total GR (range 41–65% and 42–64% respectively) and GR-D represented ∼19% (range 12–36% and 8–39%, respectively). For the 1B microvariants the GR-A levels were lower, but GR-D levels higher. Taking all the microvariable constructs together, there was a statistical significant transcript variant*protein isoform interaction (DF = 39, *F* = 3.6, *P*-value < 0.001, Two-Way ANOVA). This transcript variant*protein isoform interaction was maintained when exon microvariants were treated independently. Detailed examination of the 1A3 variant (DF = 12, *F* = 9.064, *P*-value < 0.001, Two-Way ANOVA, Figure 8A) showed that in the subsequent Student-Newman-Keuls’ pairwise comparison (Figure 8B) there was a significant difference in the level of GR-A for the different microvariable constructs, as well as GR-D and GR-B. The expression of each protein isoform was heavily influenced by the transcript length (Figure 8B). GR-A levels from the 1A3 94nt variant were significantly higher from the other 4 mRNA microvariants (Figure 8B). The published 1A3 variant (1A3 mini) has significant higher GR-D expression levels compared to all shorter microvariants, additionally its GR-C expression level was significantly lower compared to the 1A3 180nt variant (Figure 8B). Hence, the considerable differences in translational efficiency amongst the alternative *GR*-transcripts suggests that the 5′ UTR variability, and thus TSS microvariability, is involved in the modulation of transcript isoform levels and consequently in protein isoform levels.

### Evolutionary analysis of *NR3C1*

The relative conservation of both the variable upstream promoter regions and the conserved *GR* encoding regions were visually investigated from the UCSC 100 vertebrate phyloP conservation data (Figure 8 and Supplementary Data Figure S14). Conservation values within exon 2 are higher and more constant than those in the distal and proximal promoter region, where the conservation pattern was more variable (Figure 8 and Supplementary Data Figure S14). Within both the distal and proximal promoter region the microviariable TSSs did not coincide with regions of increased or decreased evolutionary conservation, but were randomly distributed. This observation was confirmed by plotting TSS usage against phyloP phylogenetic values (Figure 8C), where no trend was detected. The bulk of the TSSs, irrespectively of their frequency, were centred within the phylogenetic *P*-value range from −2 till 2, suggesting that irrespective of their frequency, most TSSs were relatively neutral concerning evolution rates, the conservation of these sites is not high, nor is their evolution rate. Throughout the distal and proximal promoter the phyloP conservation level was distributed around zero (Figure 8D). Although the coding exon 2 has previously been reported to be highly conserved (41), this was not so. The transcription factor binding sites identified by in silico phylogenetic footprinting (38) did not coincide with nucleotide position of the differentially expressed TSSs (Figure 8E). Overall it seems that the observed human TSS microvariability does not occur in regions that are evolutionary conserved, suggesting that the pattern of microvariable TSS usage may be species specific.

## DISCUSSION

We detected a variability in TSS location that has so far received little attention and that appears to be due to a highly permissive transcriptional machinery. The combination of 5′ mRNA cap labelling and NGS enabled us to identify clusters of TSSs (loci) consisting of 4–10 adjacent microvariable TSSs. The simple mono-exonic *ADR2BR* utilised a single locus consisting of four adjacent TSSs. The multi-promoter *GR* gene targeted 358 TSSs distributed throughout 38 contiguous loci. We observed TSSs with frequencies from 6.74 × 10^−4^% to 38.5% of the total 5′ m^7^G transcripts. This expands previous reports from ourselves and others of multiple alternative first exons that temporally, spatially, and quantitatively regulate mRNA levels and isoforms (11,16,17,21,25,42–44), suggesting an almost unlimited transcriptional variability. This transcriptional microvariability had a significant effect on the relative abundance of the final *GR* N-terminal translational isoforms.

The experimental protocol was carefully designed to exclude other potential interpretation of these NGS results. Albeit mRNA extraction and 5′ labelling were performed in the presence of RNase inhibitors, it would be conceivable that the 5′ m^7^G cap and adjacent nt may have been removed by residual RNase activity leading to an apparent ragged TSS pattern. This potential artefact was essentially excluded by the RNA oligo ligation strategy. First, CIP dephosphorylation removed all active 5′ mono-phosphates from truncated or otherwise degraded mRNA as well as all other RNAs, leaving only intact capped mRNA unaffected. Subsequently, TAP pyrophosphatase treatment hydrolyses the pyrophosphate bonds in the m^7^G cap triphosphate bridge leaving only mature, undegraded mRNAs with a 5′ mono-phosphate that is available for oligo ligation (30–32). Thus the combination of CIP and TAP exclusively labels 5′ m^7^G capped sequences (45). The enzymatic mechanisms of both enzymes are well known from their high resolution crystal structures. The latter studies clearly demonstrated RNA binding and formally exclude exonuclease or phosphodiesterase activity (46). Indeed, 5′-RACE is a well-established technique and incorrect identification of the 5′ m^7^G capped TSSs has not been previously reported (30–32,45,47,48). To further reduce the possibility of artefacts due to high sequencing depth, we introduced a 0.1% cut-off to define a genuine TSS. This corresponds to the point of inflection on the frequency/cut-off curve, the point where the phases of the linear regression intersect, above which the numbers of identified TSSs did not significantly decrease (Supplementary Data, Figures S15 and S16).

Additionally, our experimental protocol was also designed to minimise any potential ligation bias. Both RNA and DNA ligases exhibit intrinsic sequence preferences, particularly with respect to the 3′ end of the ligated 3′-5′ pair (49–55). By using a common RNA or DNA oligo with a constant 3′ end, we effectively minimised the ligation bias within- and between- samples. Yet sample-specific MID usage, ligating a variable 3′ end to the common 5′ end of the template, would still cause ligase induced bias to affect multiplex sequencing experiments (49,51,53). Hence, sequencing one sample using different MIDs should not result in varying TSS usage profiles because of the common RNA-oligo (Figure 1C and D), but rather in limited differences in overall relative read numbers because of biased efficiencies during library preparation (49,50). Both *ADRB2R* and *GR* data sets were screened for ligation bias. When samples were run repeatedly with different MID sequences, no discriminating or run-specific TSS expression profiles were detected. Therefore, we conclude that errors introduced during the sample preparations are minimal and below our 0.1% cut-off. Furthermore, comparisons of sequencing replicates allowed us to distinguish between the low technical variability and high biological variability observed in unstimulated DAUDI cells. Thus our judicious experimental set-up and control experiments demonstrated that the microvariability in TSS usage is a genuine biological phenomenon. Ragged 5′ ends of mRNA transcripts so far have been reported in one keynote report in the case of the genes *Postn*, *Myh3* and *Fth*1 (1) and recently the importance of alternative 5′ end mRNA for transcriptome and proteome diversity has gained attention (5,12). As such, TSS locations may be considered exact, however, the number of sequences at each position should be considered semi-quantitative, as the minimal bias introduced during the RACE-PCR amplification can not be quantified. NGS-RACE provides an accurate gene specific view of TSS location, and as such is complimentary to genome wide techniques such as CAGE (56), ‘gene identification signatures (GIS)’ and ‘gene signature cloning (GSC)’ (57). These genome-wide techniques cover the complete transcriptional landscape at a cost of several orders of magnitude more sequencing data, whilst NGS-RACE provides greater insight, due to the greater sequencing depth it permits for single, often weakly expressed genes, as well as allowing longer sequence tags than MmeI digestion (56).

When our observations were compared to the previously published gene and mRNA structures, the simple mono-exonic gene *ADRB2R* had a small increase in complexity, from 1 TSS (19) to a locus of four adjacent TSSs. The multi-exonic *GR*, however, displayed a ∼30 fold increase in TSSs, going from nine previously published TSSs (19,21–26) to 358 in 38 loci in the absence of any specific transcriptional stimuli. 66.7% of the newly identified *GR* TSSs were located within the proximal promoter, 16.1% corresponded to the distal promoter, with the remaining 17.2% situated within exon 2. Dex and IFN-γ, both ligands of transcription factors activating the *GR* promoter further induced transcription from one new locus and 185 additional TSSs distributed throughout the promoter region, giving a ∼40-fold overall increase in the number of *GR* TSSs. These TSSs are mostly located within loci utilized also in other cell lines. Thus transcription does not seem to be initiated at a well-defined, fixed, TSS, but their selection seems to move due to a more or less permissive transcription machinery. The effect of the transcription factor ligands Dex and IFN-γ may suggest that the transcription factor complex determines the start site of the transcription. Steric effects of binding of transcription factor complexes to the promoter DNA may perhaps be the simplest explanation for TSS microvariability. Although the microvariability may appear to be to some extent stochastic, we demonstrate that, in the case of *GR*, it also regulates translation. There are several potential scenarios. When microvariable TSSs are upstream of the principal ATG translation initiation codon, the complete coding sequence (CDS) remains available in the mRNA and can be translated, without effect on the protein sequences (Supplementary Data Figure S17). Splicing to the subsequent exon was never affected and consistently performed to the common splice acceptor site. However, we observed for the *GR* that some TSSs were located downstream of the principal translation initiation codon in exon 2. These abridged mRNAs may produce N-terminally truncated protein isoforms from methionine-encoding ATG codons further downstream. Such truncated GR isoforms starting at alternative downstream translation initiation codons indeed exist, and are well known as GR-A, -B, -C and -D protein isoforms (58) (Supplementary Data Figure S17). These internal translation initiation codons are available in the full-length mRNA. In line with our previous study (11), where the alternative first exons altered mRNA folding stability, half-life, translation efficiency and protein isoform production in a length-dependent, but sequence-independent manner. We observed identical negative correlations for ΔG-mRNA length, ΔG-translational efficiency and mRNA length-translational efficiency to those in our previous report (11), suggesting that microvariability plays a similar role in the regulation of both transcript and protein levels. We were able to extend this, demonstrating that differences in TSS location of only a few nucleotides within a locus dramatically altered the fine balance between the different N-terminal *GR* isoforms. There are evermore reports of multiple active or alternative initiation codons within a mature mRNA, covering both leaky ribosome scanning and internal ribosome entry in plants (59,60) and mammals (61,62) as well as classical viral IRES. Given that internal ATG codons and methionines are ubiquitous(63,64), we suggest that our observation may apply ubiquitously throughout the transcriptome and throughout evolution. The evolution speed of genes with complex 5′ UTRs is negatively correlated with their expression level and is also dependent on functional specialization of the genes. With a high intron density being one of the characteristics associated with a slower evolution rate (10,65). We examined the evolutionary conservation of the *GR* CpG island and distal promoter region. As would be expected the non-coding regions were less highly conserved than the coding regions in exon 2. Since the microvariable TSSs did not coincide with either evolutionary conserved transcription factor binding sites or more generally regions of high conservation we suggest that whilst TSS microvariability and its functional consequences are most likely identical between species, the actual TSSs selected will be species specific. These minor changes in TSS dramatically altering the protein isoforms produced and their function may underlay the vastly inflated proteome, significantly increasing the variability from the limited genome to the proteome (5,11–15). The microvariable transcription initiation is an additional mechanism in the spectrum of alternative transcription initiation mechanisms.

The demethylation agent AZA also had a profound effect on TSS usage. Complete de-methylation with AZA induced 12 specific TSSs and another 115 that were also observed for either Dex- or IFN-γ- treated DAUDI cells or one of the other cell lines. One new locus was identified. In a similar manner to Dex and IFN- γ treatment, demethylation will alter the balance between the different *GR* translational isoforms. Additionally, the vast increase in the overall number of TSSs raises doubts over the functional consequences of single CpG dinucleotide methylation for the regulation of *GR* expression (22,66–68). We would, however, anticipate that increased methylation levels over a larger cluster of CpG dinucleotides will influence total GR levels by silencing one or more loci, concordant with our observations of methylation clusters in several models (25,69). This raises the interesting hypothesis that the epigenetically controlled response to GC, as previously observed (24–27,70,71) is due to DNA methylation influencing TSS usage and altering the balance between translational isoforms. There are examples of internal methionine encoding ATGs also serving as secondary translation initiation sites in many vertebrate, invertebrate and plant species (59–62), making them amendable to TSS microvariability induced differential translation initiation.

In conclusion, we suggest that our observations of permissive microvariable transcription may also be expanded to other genes since many possess a structure similar to the *GR* (4–6,10,12,16,17,42,72,73). Our observations further suggest that TSS microvariability is not simply the result of a permissive transcription machinery, but rather a mechanism to fine-tune total protein levels via multiple mRNAs species that differ in stability and in some cases the relative distribution of protein isoforms. We showed that TSS usage can be influenced by transcription factor ligands. Similarly DNA methylation seems to influence TSS selection, adding another mechanism by which covalent modifications of DNA can regulate gene expression to match physiological requirements.

## DATA AVAILABILITY

The Ion Torrent data have been deposited in the European Nucleotide Archive (ENA) of the EMBL-EBI under accession number PRJEB9064

## Supplementary Material

SUPPLEMENTARY DATA
