# Supplementary material for: Where does transcription start? 5′-RACE adapted to next-generation sequencing
Source: Nucleic Acids Res. 2015 Nov 28;44(6):2628–45. doi: 10.1093/nar/gkv1328 (PMC4824077; doi:10.1093/nar/gkv1328)
Supplement: SUPPLEMENTARY DATA [file supp_44_6_2628__index.html]

Where does transcription start? 5′-RACE adapted to next-generation sequencing — Where does transcription start? 5′-RACE adapted to next-generation sequencing — SUPPLEMENTARY DATA 

# Where does transcription start? 5′-RACE adapted to next-generation sequencing

## SUPPLEMENTARY DATA

- SUPPLEMENTARY DATA
