# Supplementary material for: Where does transcription start? 5′-RACE adapted to next-generation sequencing
Source: Nucleic Acids Res. 2015 Nov 28;44(6):2628–45. doi: 10.1093/nar/gkv1328 (PMC4824077; doi:10.1093/nar/gkv1328)
Supplement: SUPPLEMENTARY DATA [file supp_gkv1328_nar-01103-z-2015-File010.pdf]

Table 1: PCR primers

Primer name	Sequence
GeneRacer 5' primer_fwd	5'-GACTGGAGCACGAGGACACTGA-3'
Exon 2_1st PCR deep seq_rev	5‘-GGAACACTGGTCGACCTATTGAGGT-3‘
ADRB2R_1st PCR_deep seq_rev	5'-CTTCCATTGGGTGCCAGCAAG-3‘
GeneRacer 5' nested_fwd	5‘-GGACACTGACATGGACTGAAGGAGTA-3‘
Exon 2_2nd PCR deep seq_rev	5‘-GGAGTCTGATTGAGAAGCGACAGC-3‘
ADRB2R_2nd PCR_deep seq_rev	5'-CTCATTCAGCGGCTGTGGTG-3‘

Table 2: Plasmid variants for translational efficiency analysis

Plasmid Name	Length Insert (nt)	Sequence Insert (5' – 3')
CMV-1A3	94	AGTTGTACCTTAATAACAGGAATTTTCATCTGCCTGGCTCCTTTCCTCAAAGAACAAAGAAG<br>ACTTTGCTTCATTAAAGTGTCTGAGAAGGAAG
CMV-1A3	160	AATGAGTGCCTTCTCTGTGCGAGAATGGGGAGGAACAAAATGCAGCTCCTACCCTCCTCGG<br>GCTTTAGTTGTACCTTAATAACAGGAATTTTCATCTGCCTGGCTCCTTTCCTCAAAGAACAAA<br>GAAGACTTTGCTTCATTAAAGTGTCTGAGAAGGAAG
CMV-1A3	164	ACTTAATGAGTGCCTTCTCTGTGCGAGAATGGGGAGGAACAAAATGCAGCTCCTACCCTCC<br>TCGGGCTTTAGTTGTACCTTAATAACAGGAATTTTCATCTGCCTGGCTCCTTTCCTCAAAGAA<br>CAAAGAAGACTTTGCTTCATTAAAGTGTCTGAGAAGGAAG
CMV-1A3	180	ATTAACTTTGATAAGCACTTAATGAGTGCCTTCTCTGTGCGAGAATGGGGAGGAACAAAAT<br>GCAGCTCCTACCCTCCTCGGGCTTTAGTTGTACCTTAATAACAGGAATTTTCATCTGCCTGGC<br>TCCTTTCCTCAAAGAACAAAGAAGACTTTGCTTCATTAAAGTGTCTGAGAAGGAAG
CMV-1A3	981	AGGTTATGTAAGGGTTTGCTTTCACCCCATTCAAAAGGTACCTCTTCCTCTTCTCTTGCTCCC<br>TCTCGCCCTCATTCTTGTGCCTATGCAGACATTTGAGTAGAGGCGAATCACTTTCACTTCTGC<br>TGGGGAAATTGCAACACGCTTCTTTAAATGGCAGAGAGAAGGAGAAAACTTAGATCTTCTG<br>ATACCAAATCACTGGACCTTAGAAGGTCAGAAATCTTTCAAGCCCTGCAGGACCGTAAAAT<br>GCGCATGTGTCCAACGGAAGCACTGGGGCATGAGTGGGGAAGGAATAGAAACAGAAAGA<br>GGGTAAGAGAAGAAAAAAGGGAAAGTGGTGAAGGCAGGGAGGAAAATTGCTTAGTGTG<br>AATATGCACGCATTCATTTAGTTTTCAAATCCTTGTTGAGCATGATAAAATTCCCAGCATCAG<br>ACCTCACATGTTGGTTTCCATTAGGATCTGCCTGGGGGAATATCTGCTGAATCAGTGGCTCT<br>GAGCTGAACTAGGAAATTCACCATAATTAGGAGAGTCACTGTATTTCTCTCCAAAAAAAAA<br>AAAGTTATACCCGAGAGACAGGATCTTCTGATCTGAAATTTTCTTCACTTCTGAAATTCTCTG<br>GTTTGTGCTCATCGTTGGTAGCTATTTGTTCATCAAGAGTTGTGTAGCTGGCTTCTTCTGAA

		AAAAGGAATCTGCGTCATATCTAAGTCAGATTTCATTCTGGTGCTCTCAGAGCAGTTAGCCC<br>AGGAAAGGGGCCAGCTTCTGTGACGACTGCTGCAGAGGCAGGTGCAGTTTGTGTGCCACA<br>GATATTAACTTTGATAAGCACTTAATGAGTGCCTTCTCTGTGCGAGAATGGGGAGGAACAA<br>AATGCAGCTCCTACCCTCCTCGGGCTTTAGTTGTACCTTAATAACAGGAATTTTCATCTGCCT<br>GGCTCCTTTCCTCAAAGAACAAAGAAGACTTTGCTTCATTAAAGTGTCTGAGAAGGAAG
CMV-1B	53	AGATGATGCGGTGGTGGGGGACCTGCCGGCACGCGACTCCCCCCGGGCCCAAA
CMV-1B	73	AGCTGAAGACCCGGCCGCCCAGATGATGCGGTGGTGGGGGACCTGCCGGCACGCGACTCC<br>CCCCGGGCCCAAA
CMV-1B	107	AACTTCTCTCCCAGTGCGAGAGCGCGGCGGCGGCAGCTGAAGACCCGGCCGCCCAGATGA<br>TGCGGTGGTGGGGGACCTGCCGGCACGCGACTCCCCCCGGGCCCAAA
CMV-1B	105	CTTCTCTCCCAGTGCGAGAGCGCGGCGGCGGCAGCTGAAGACCCGGCCGCCCAGATGATG<br>CGGTGGTGGGGGACCTGCCGGCACGCGACTCCCCCCGGGCCCAAA
CMV-1C	71	AAGCTAAGTTGTTTATCTCGGCTGCGGCGGGAACTGCGGACGGTGGCGGGCGAGCGGCTC<br>CTCTGCCAGAG
CMV-1C	73	ACAAGCTAAGTTGTTTATCTCGGCTGCGGCGGGAACTGCGGACGGTGGCGGGCGAGCGGC<br>TCCTCTGCCAGAG
CMV-1C	101	ATATTTCCCTCCTGCTCCTTCTGCGTTCACAAGCTAAGTTGTTTATCTCGGCTGCGGCGGGA<br>ACTGCGGACGGTGGCGGGCGAGCGGCTCCTCTGCCAGAG
CMV-1C	479	GGCGCCGCCTCCACCCGCTCCCCGCTCGGTCCCGCTCGCTCGCCCAGGCCGGGCTGCCCTTT<br>CGCGTGTCCGCGCTCTCTTCCCTCCGCCGCCGCCTCCTCCATTTTGCGAGCTCGTGTCTGTGA<br>CGGGAGCCCGAGTCACCGCCTGCCCGTCGGGGACGGATTCTGTGGGTGGAAGGAGACGC<br>CGCAGCCGGAGCGGCCGAAGCAGCTGGGACCGGGACGGGGCACGCGCGCCCGGAACCTC<br>GACCCGCGGAGCCCGGCGCGGGGCGGAGGGCTGGCTTGTCAGCTGGGCAATGGGAGACT<br>TTCTTAAATAGGGGCTCTCCCCCCACCCATGGAGAAAGGGGCGGCTGTTTACTTCCTTTTTT<br>TAGAAAAAAAAAATATATTTCCCTCCTGCTCCTTCTGCGTTCACAAGCTAAGTTGTTTATCTC<br>GGCTGCGGCGGGAACTGCGGACGGTGGCGGGCGAGCGGCTCCTCTGCCAGAG

Suppl. Data Figure 1

A. ***ADRB2R***

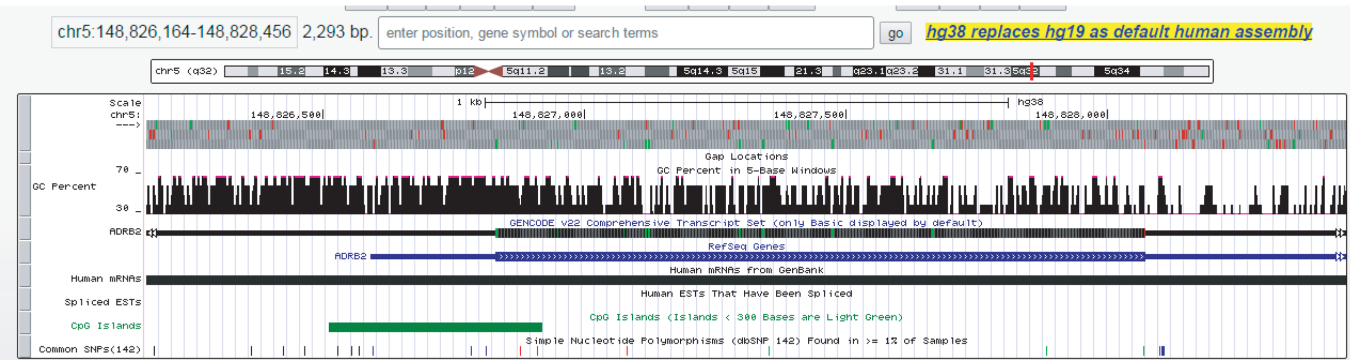

B. ***NR3C1***

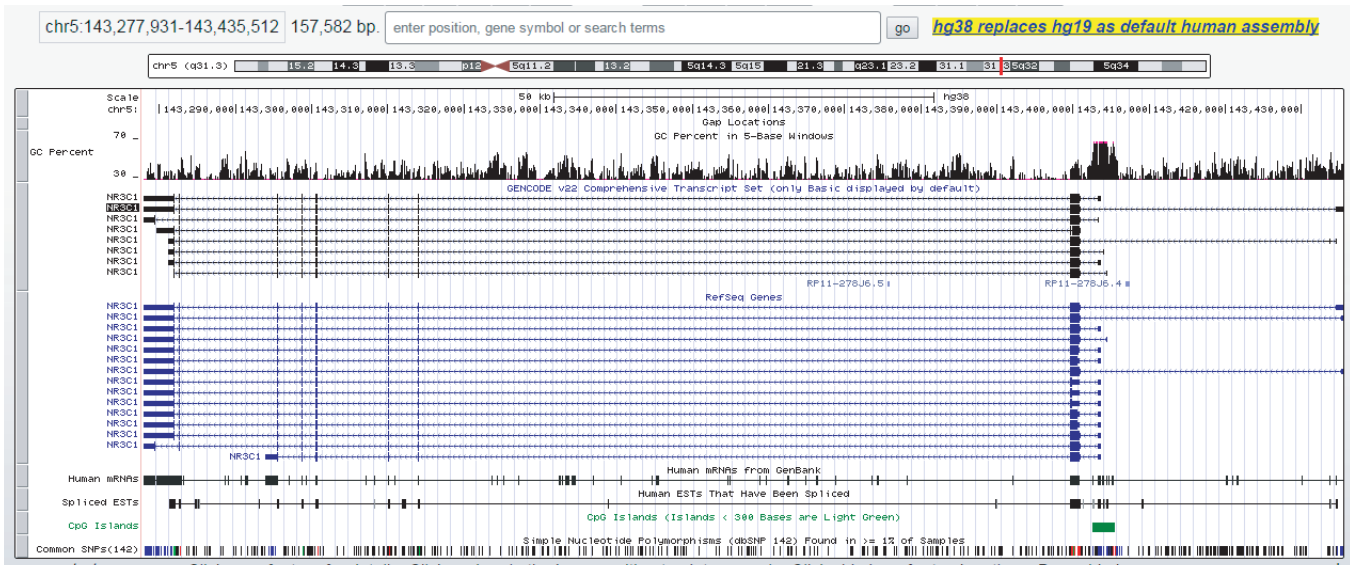

Supplementary Figure 1. (A) UCSC browser view of ADRB2R gene. (B) UCSC browser view of NR3C1 gene, showing the first exons. 1

Suppl. Data Figure 2

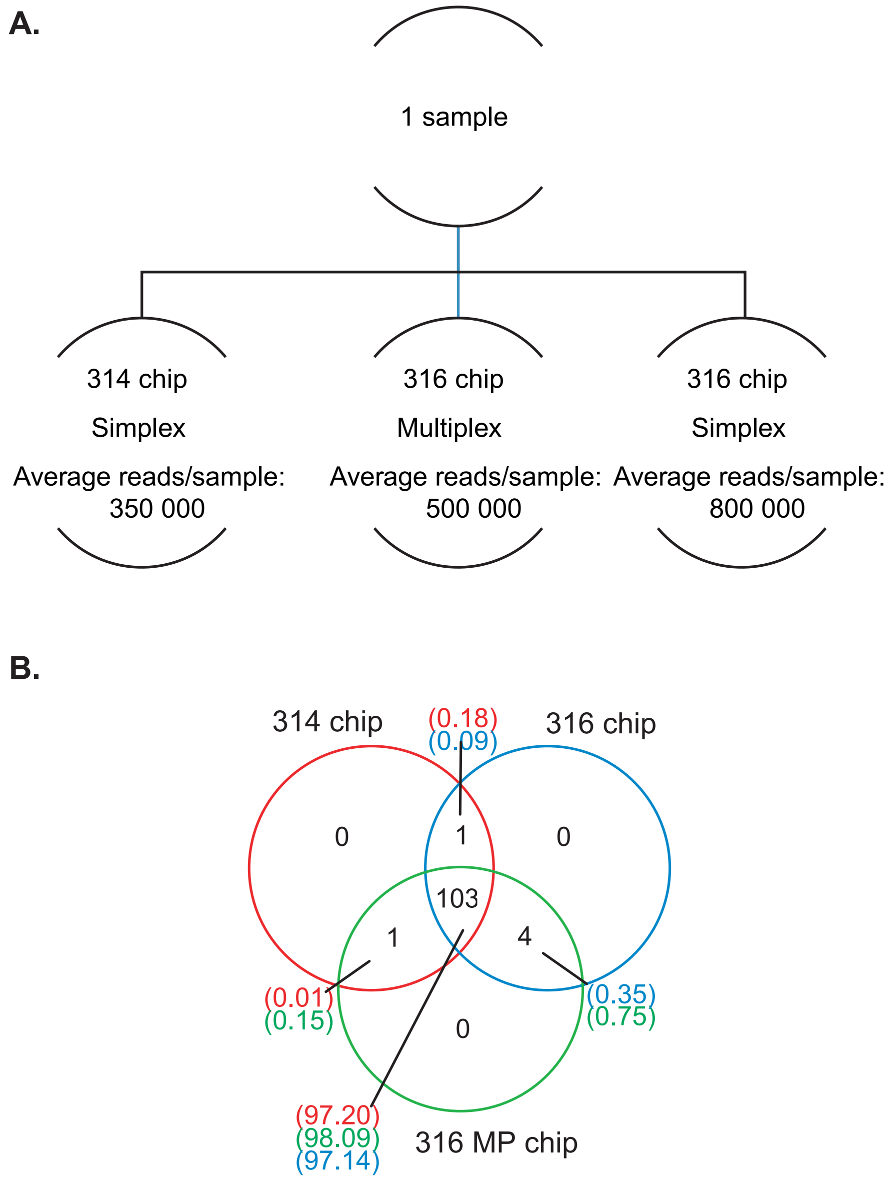

Supplementary Figure 2. (A) Over Multiplexing strategy used with Ion Torrent 314 and 316 chips. (B) The proximal NR3C1 CpG island TSSs used in three DAUDI technical replicates are plotted showing common and unique TSSs. The number of TSSs shared between DAUDI technical replicates (numbers in parentheses are the % of total labelled 5’ TSSs).

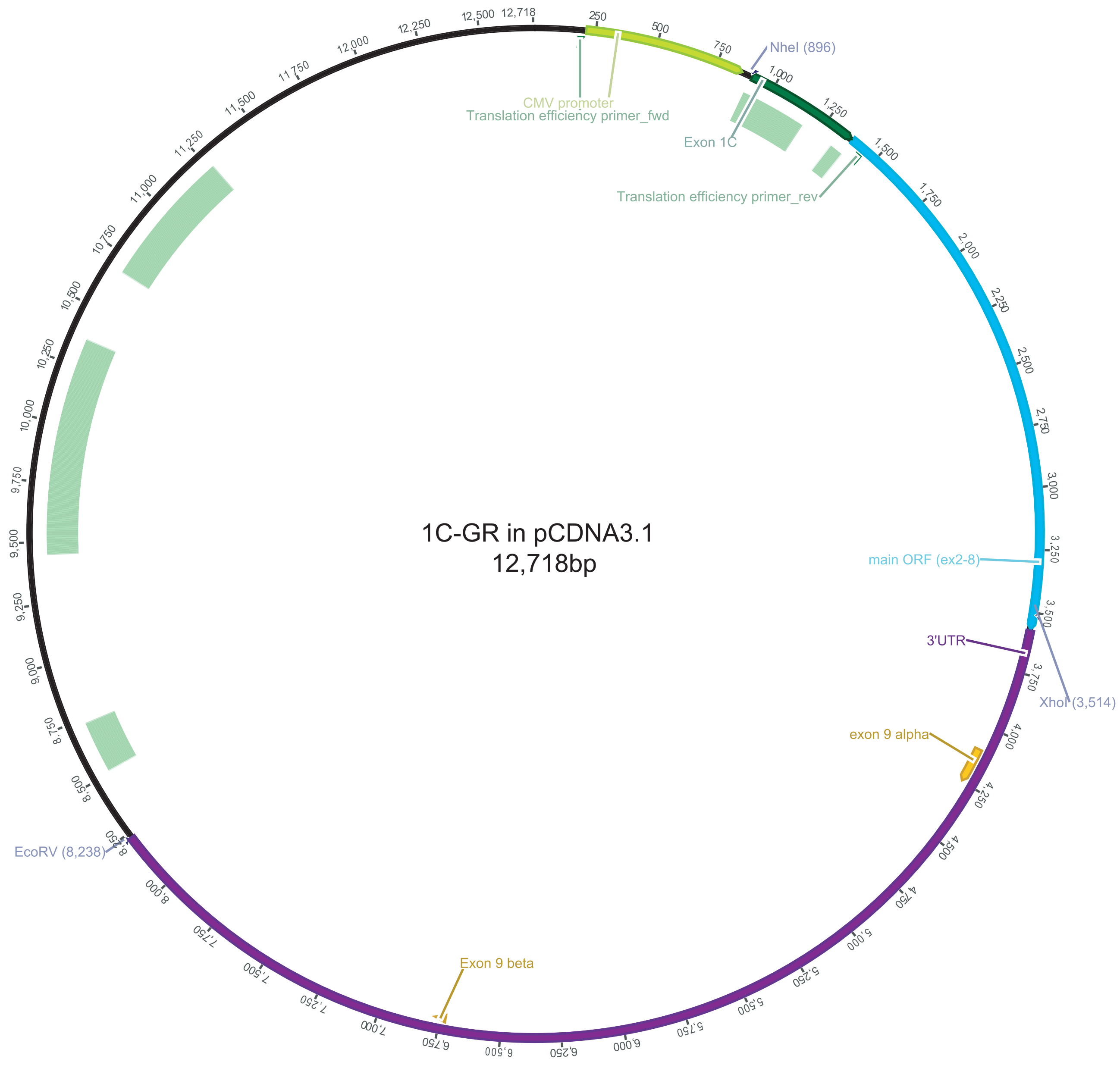

Supplementary Figure 3. The plasmid 1C GRpcDNA3.1, into which all inserts were cloned.

Suppl. Data Figure 4

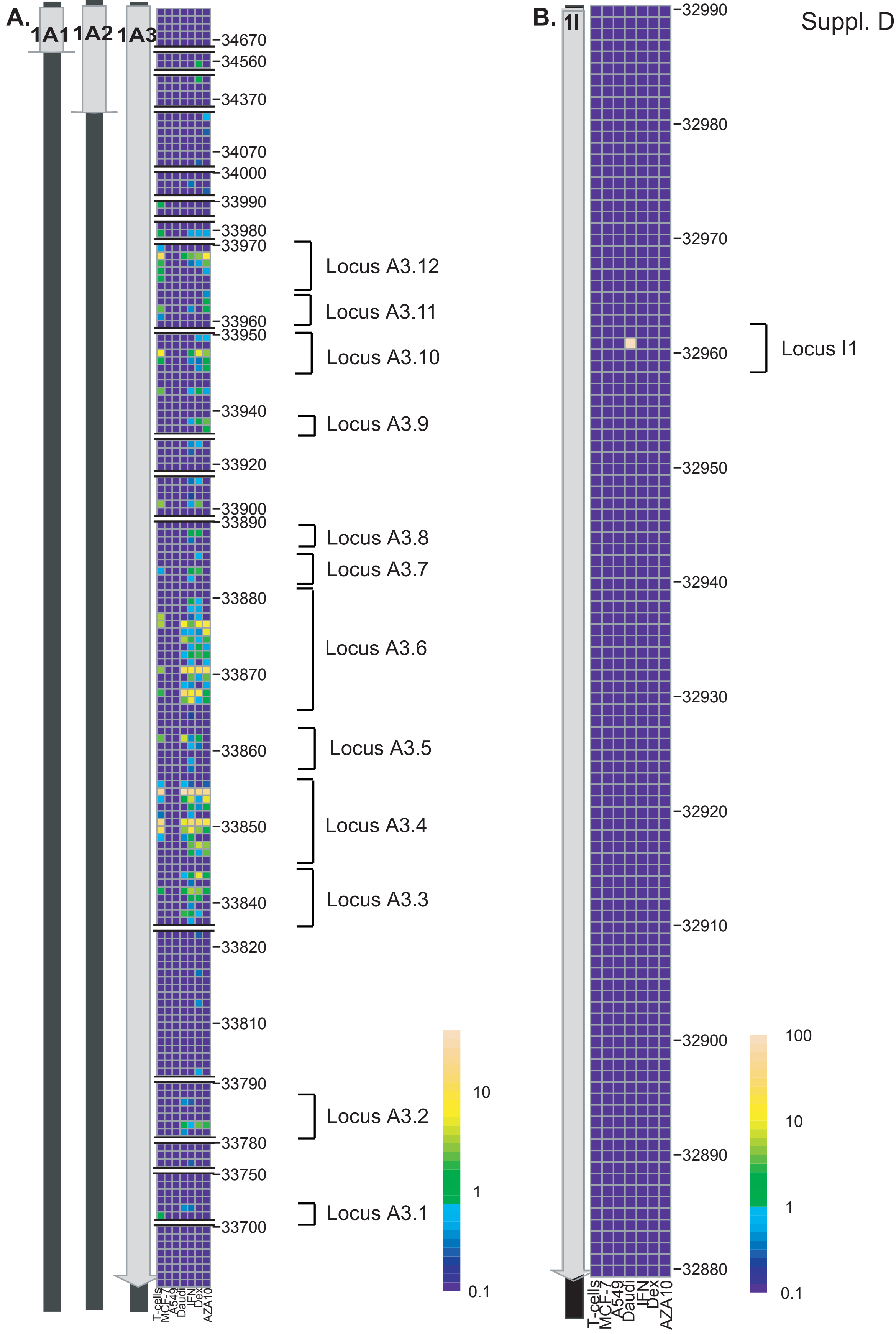

Supplementary Figure 4. Detailed TSS usage pattern for the four cell lines and three treatment condition for exon 1A and exon 1I. Published exon locations (Breslin et al, 2001; Turner and Muller 2005; Presul et al, 2007) are shown as a grey arrow, on the left. TSS usage is expressed by colour [0.1% (blue) to 100% (yellow)] logarithmically as the percentage of exon specific transcripts.

Suppl. Data Figure 5

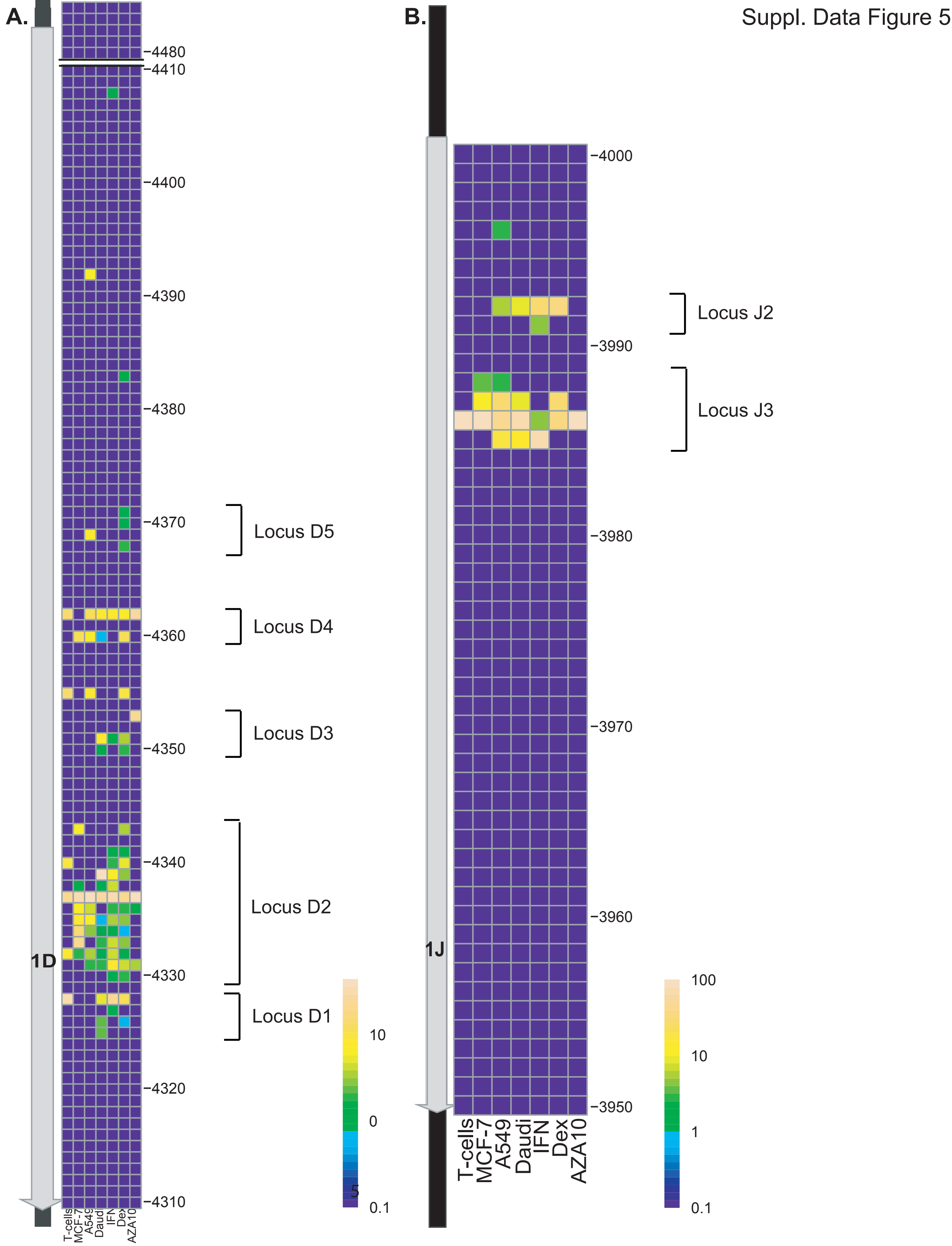

Supplementary Figure 5. Detailed TSS usage pattern for the four cell lines and three treatment condition for exon 1D and exon 1J. Published exon locations (Turner and Muller 2005) are shown as a grey arrow, on the left. TSS usage is expressed by colour [0.1% (blue) to 100% (yellow)] logarithmically as the percentage of exon specific transcripts.

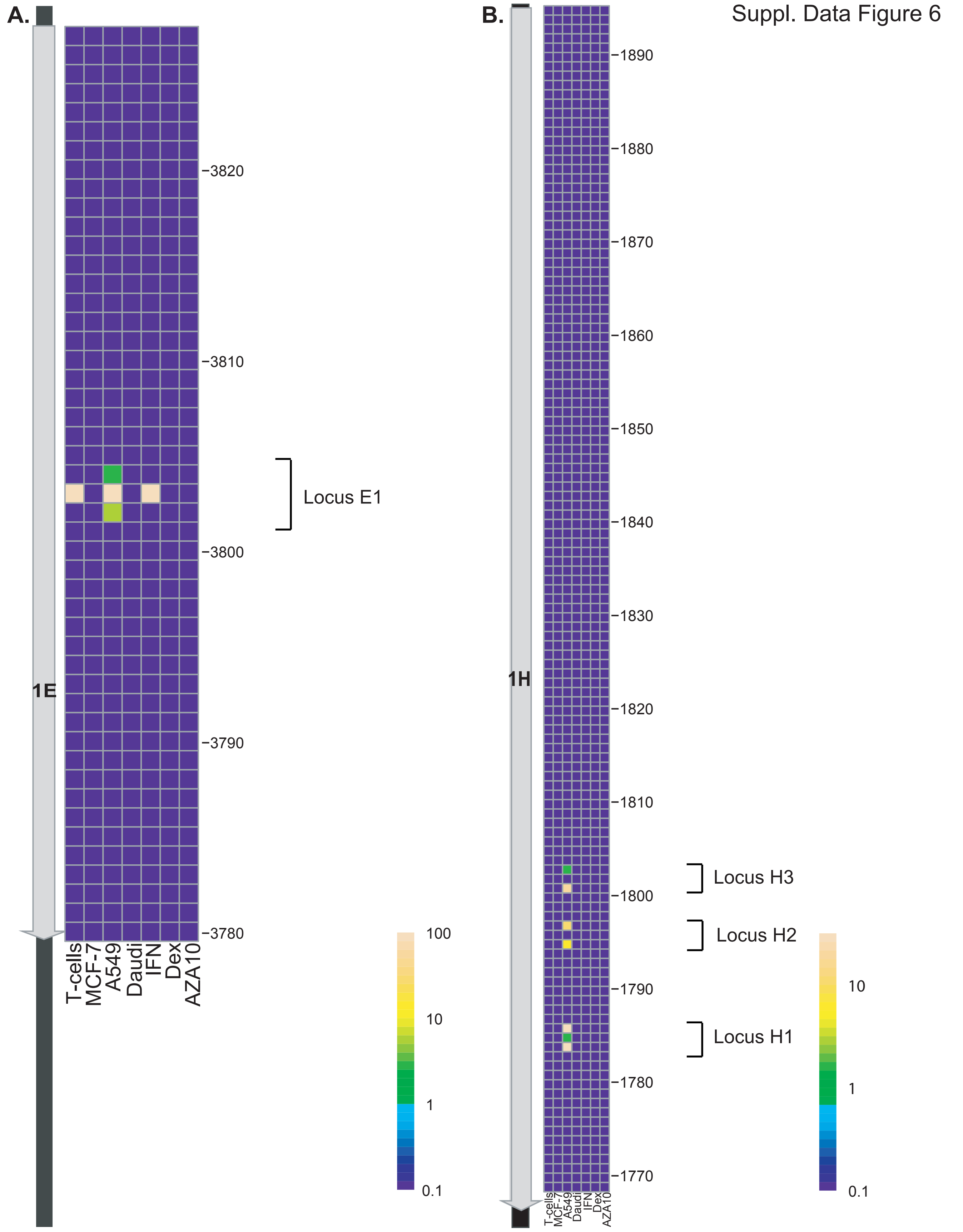

Supplementary Figure 6. Detailed TSS usage pattern for the four cell lines and three treatment condition for exon 1E and exon 1H. Published exon locations (Turner and Muller 2005) are shown as a grey arrow, on the left. TSS usage is expressed by colour [0.1% (blue) to 100% (yellow)] logarithmically as the percentage of exon specific transcripts.

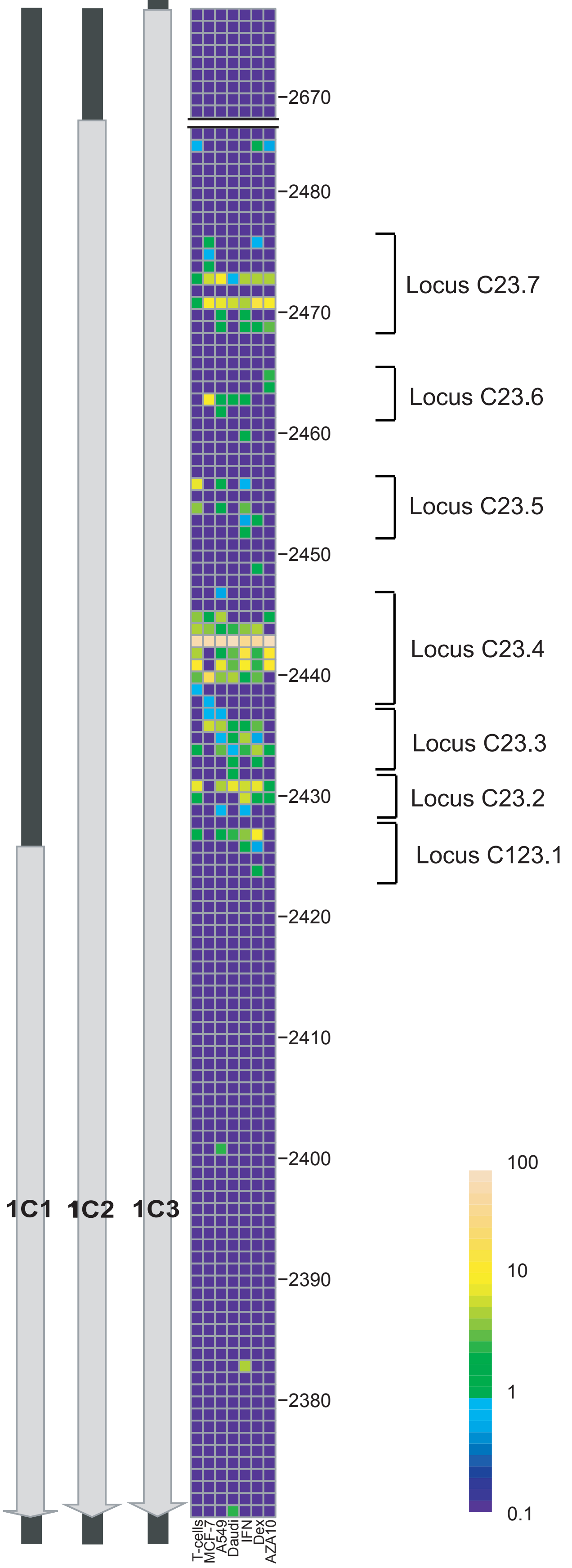

Supplementary Figure 7. Detailed TSS usage pattern for the four cell lines and three treatment condition for exon 1C. The published exon location (Turner and Muller 2005) as shown as a grey arrow, on the left. The TSS usage colour gradient is the log value of the percentage of TSS expression for exon 1C.

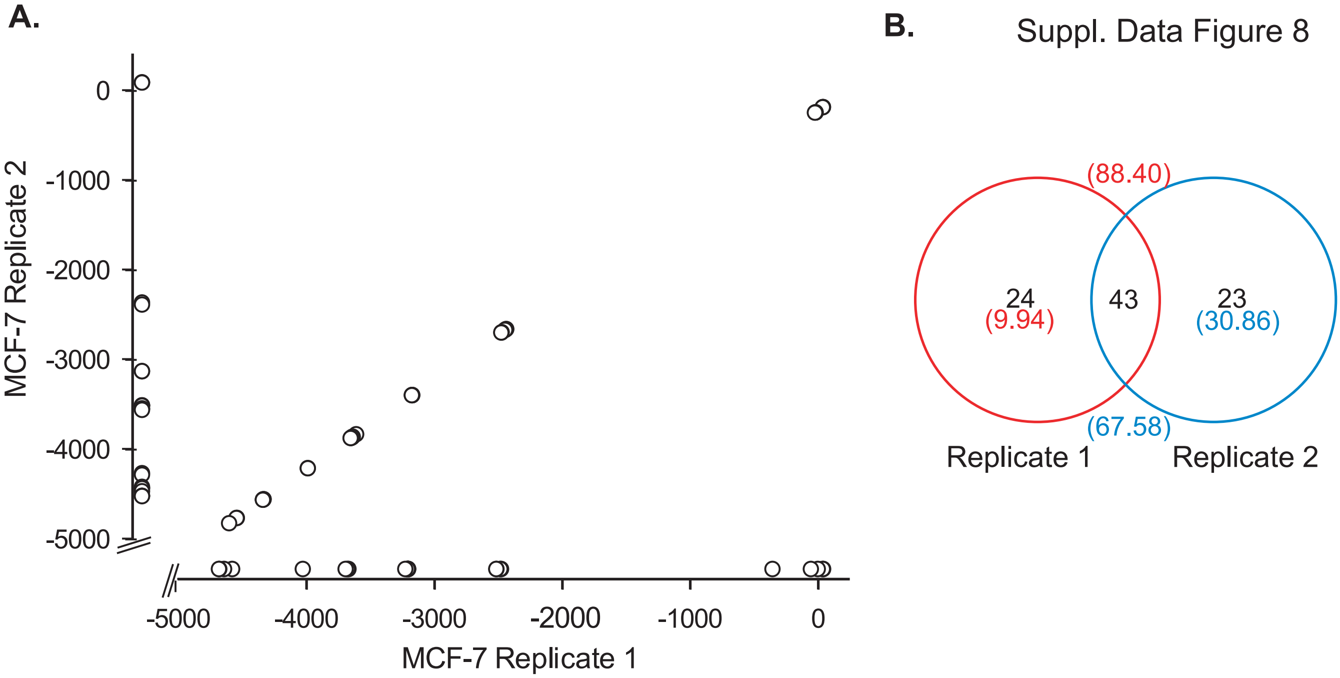

Supplementary Figure 8. Microvariable TSS distribution throughout the NR3C1 (A) The proximal NR3C1 CpG island TSSs used in two MCF-7 biological replicates are plotted showing common and unique TSSs. The NR3C1 TSSs on both axes are annotated with respect to the ATG (±1) translation initiation codon in exon 2. (B) The number of TSSs shared between MCF-7 biological replicates (numbers in parentheses are the % of total labelled 5' TSSs).

A.

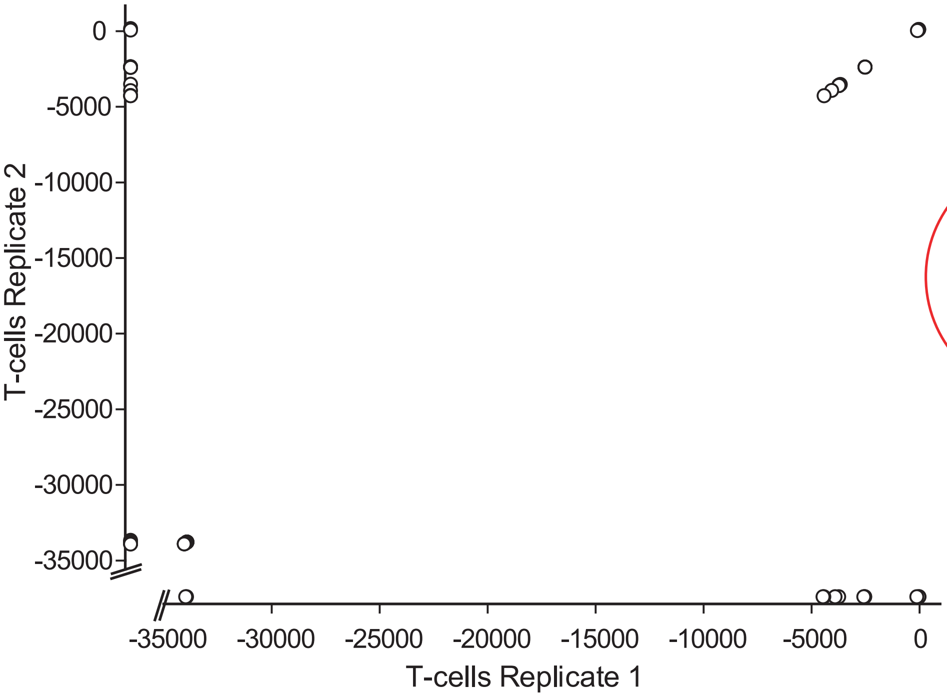

B. Suppl. Data Figure 9

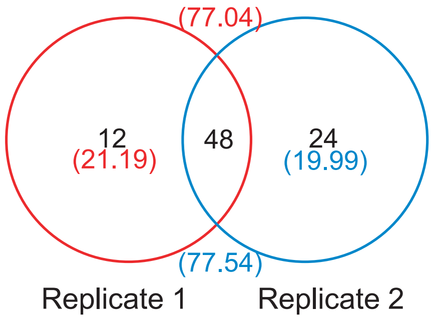

Supplementary Figure 9. Microvariable TSS distribution throughout the NR3C1 (A) The proximal NR3C1 CpG island TSSs used in two T-cells biological replicates are plotted showing common and unique TSSs. The NR3C1 TSSs on both axes are annotated with respect to the ATG (±1) translation initiation codon in exon 2.
(B) The number of TSSs shared between T-cells biological replicates (numbers in parentheses are the % of total labelled 5' TSSs).

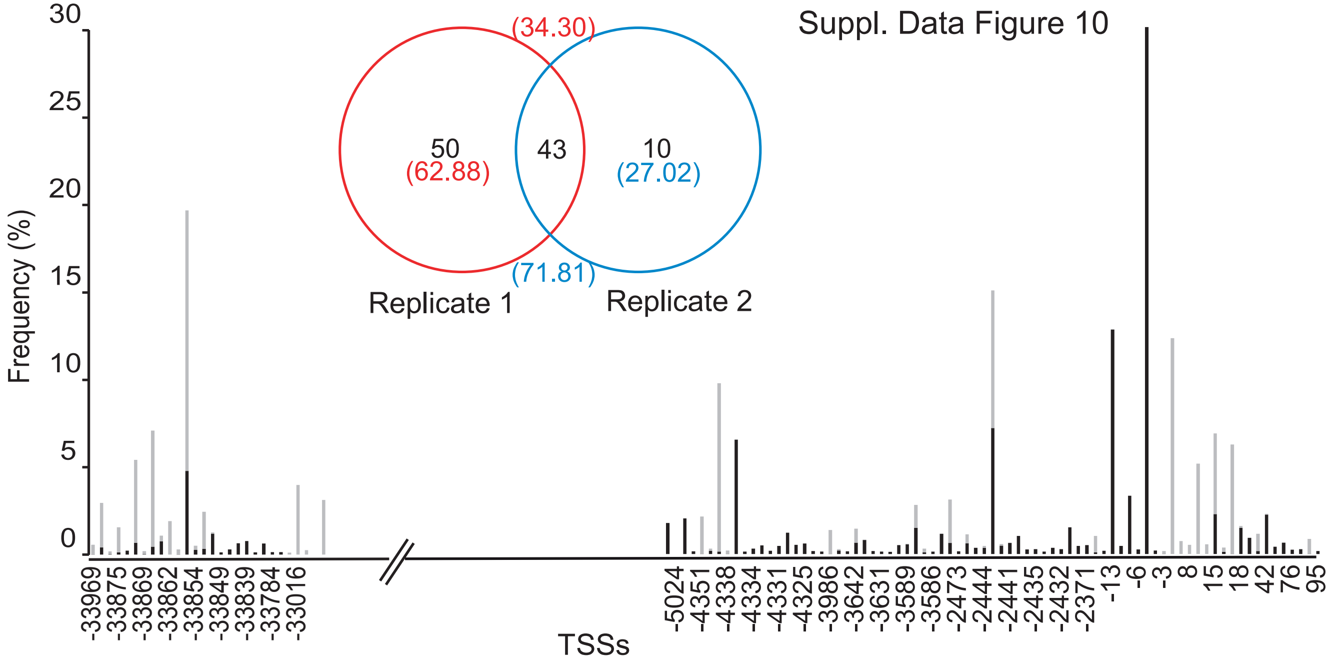

Supplementary Figure 10. The proximal NR3C1 CpG island TSSs used in two DAUDI biological replicates are plotted showing common and unique TSSs and their frequencies. The NR3C1 TSSs on the horizontal axis are annotated with respect to the ATG (±1) translation initiation codon in exon 2. The number of TSSs shared between DAUDI biological replicates (numbers in parentheses are the % of total labelled 5' TSSs).

Suppl. Data Figure 11

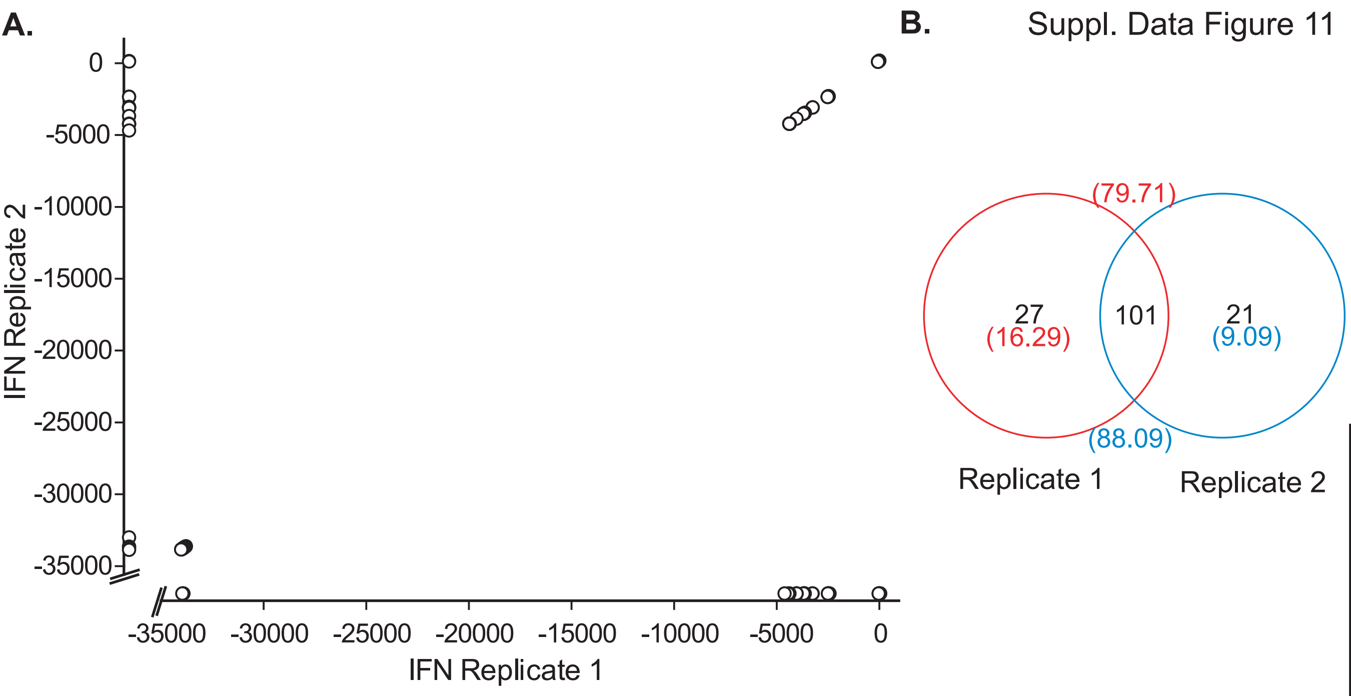

Supplementary Figure 11. Microvariable TSS distribution throughout the NR3C1 (A) The proximal NR3C1 CpG island TSSs used in two IFN-γ treated DAUDI biological replicates are plotted showing common and unique TSSs. The NR3C1 TSSs on both axes are annotated with respect to the ATG (±1) translation initiation codon in exon 2. (B) The number of TSSs shared between IFN-γ treated DAUDI biological replicates (numbers in parentheses are the % of total labelled 5’ TSSs).

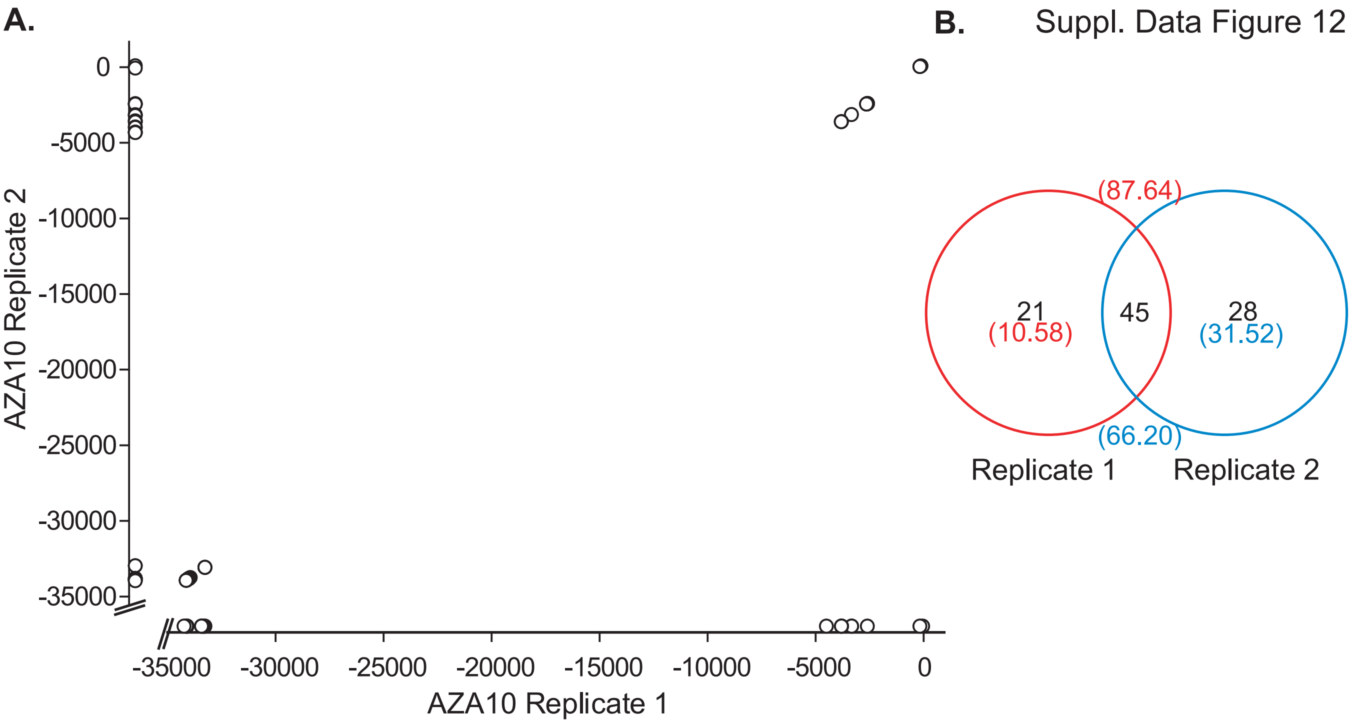

Supplementary Figure 12. Microvariable TSS distribution throughout the NR3C1 (A) The proximal NR3C1 CpG island TSSs used in two AZA10 treated DAUDI biological replicates are plotted showing common and unique TSSs. The NR3C1 TSSs on both axes are annotated with respect to the ATG (±1) translation initiation codon in exon 2. (B) The number of TSSs shared between AZA10 treated DAUDI biological replicates (numbers in parentheses are the % of total labelled 5' TSSs).

A.

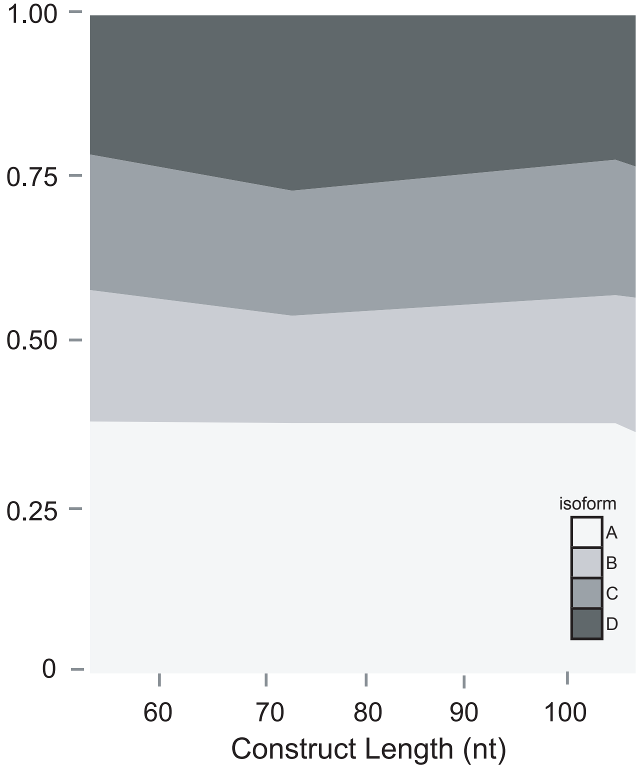

B.

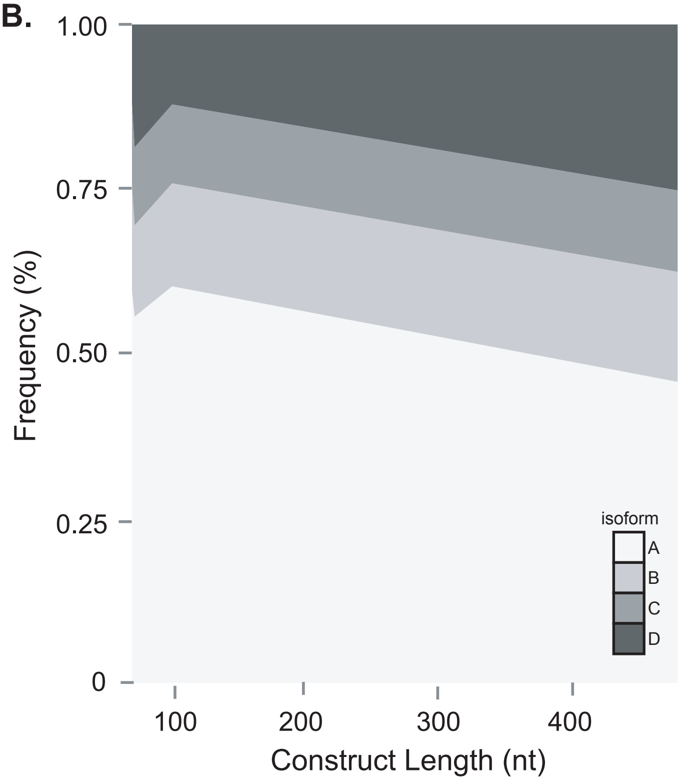

Supplementary Figure 13. The microvariable TSSs influence the relative abundance of N-terminal protein isoform. (A) Areaplot for 1B microvariable constructs, indicating the shift of isoform levels according to the construct length. (B) Areaplot for 1C microvariable constructs, indicating the shift of isoform levels according to the construct length.

Suppl. Data Figure 14

A. chr5:142,813,319-142,814,536 | 1,218 bp

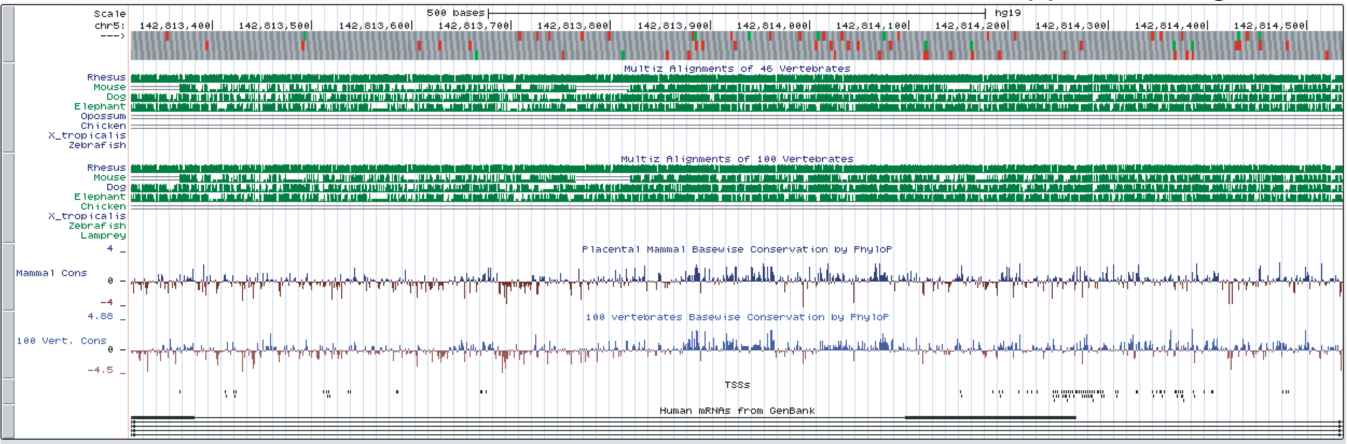

B. chr5:142,780,406-142,785,463 | 5,058 bp

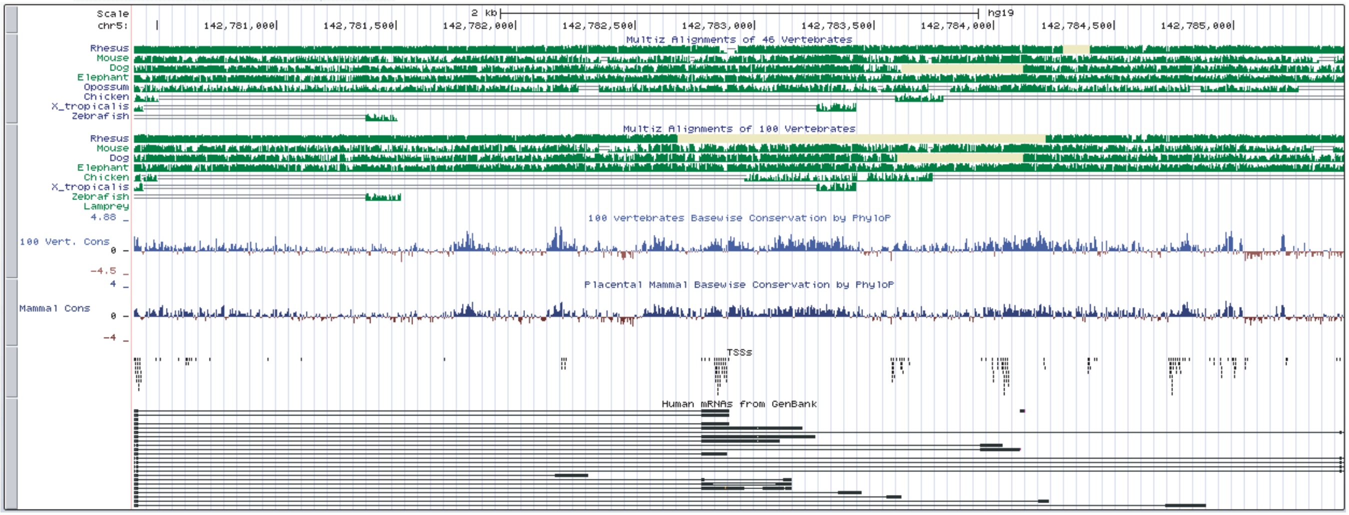

C. chr5:142,780,136-142,780,663 | 528 bp

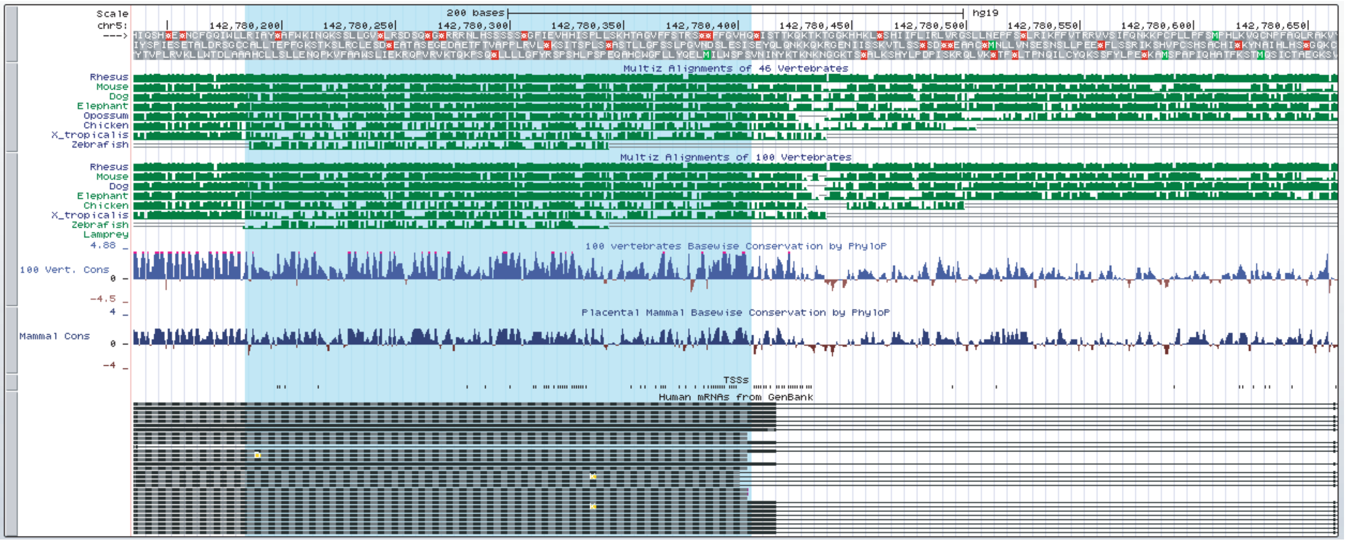

Supplementary Figure 14. Evolutionary conservation analysis of the GR’s 5’UTR using the UCSC browser. (A) The distal promoter region covering exon 1A. (B) The proximal promoter region containing the CpG island. (C) The end of the proximal promoter region and start of the common exon 2 (blue).

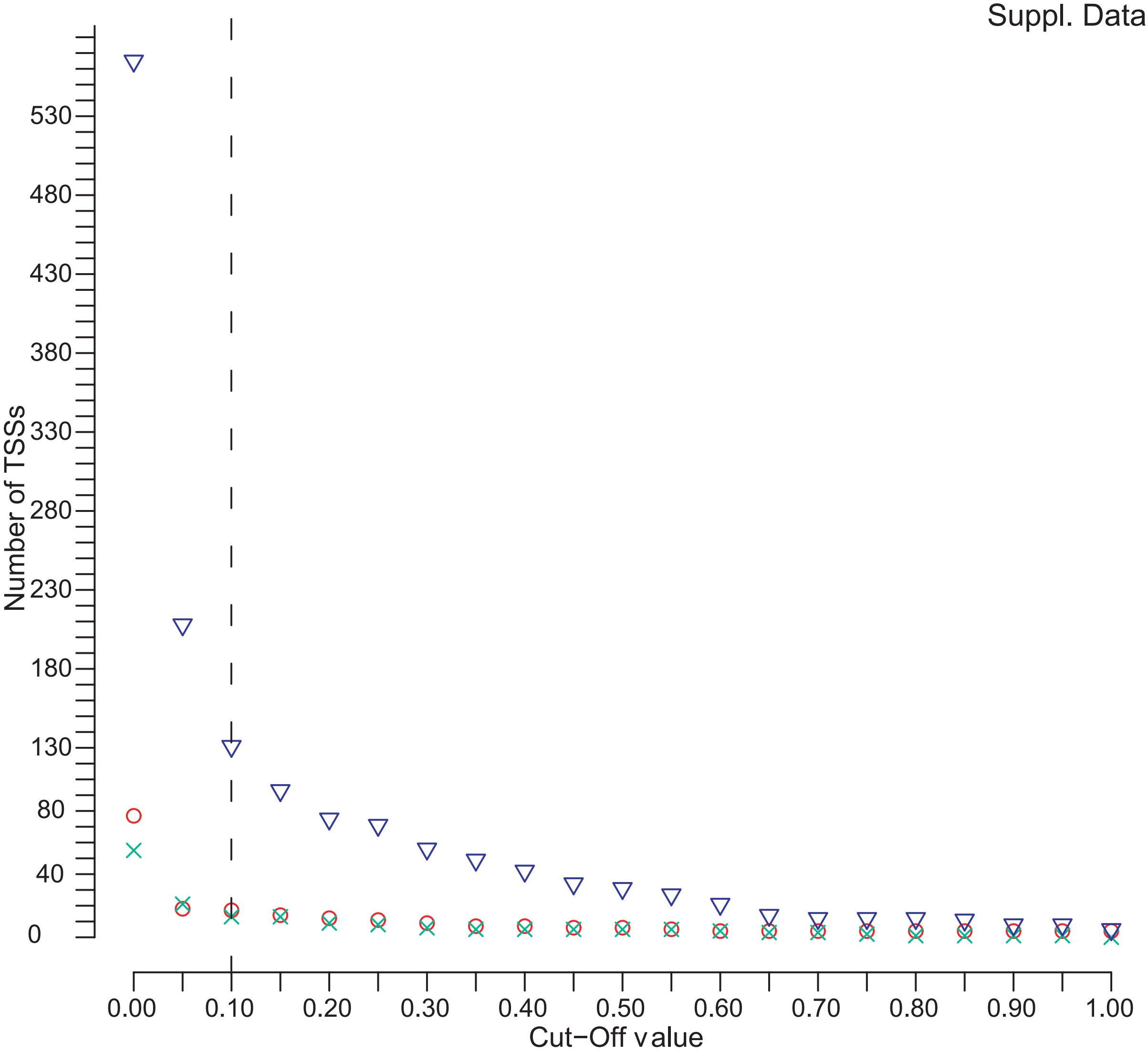

Supplementary Figure 15. The total number of identified TSSs from the NR3C1 proximal CpG island are plotted as a function of the cut-off value (%) used to define a genuine TSS. Data are from A549 biological triplicates (Run 1, **o**; Run 2, **x**; Run 3, ▽)

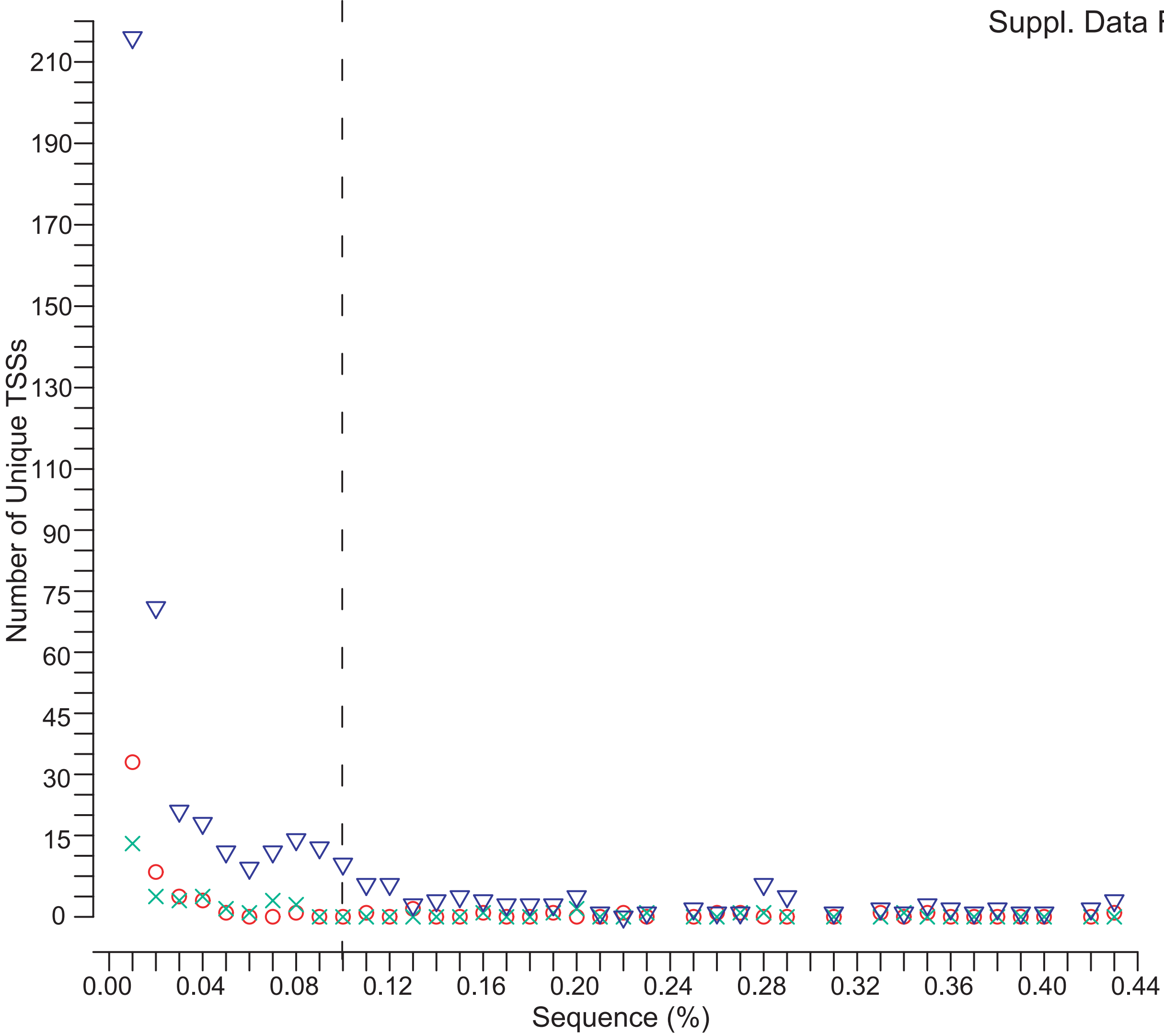

Supplementary Figure 16. The number of unique replicate specific TSSs from the NR3C1 proximal CpG island are plotted as a function of the cut-off value (%) used to define a genuine TSS. Data are from A549 biological triplicates (Run 1, **o**; Run 2, **x**; Run 3, ▽)

Suppl. Data Figure 17

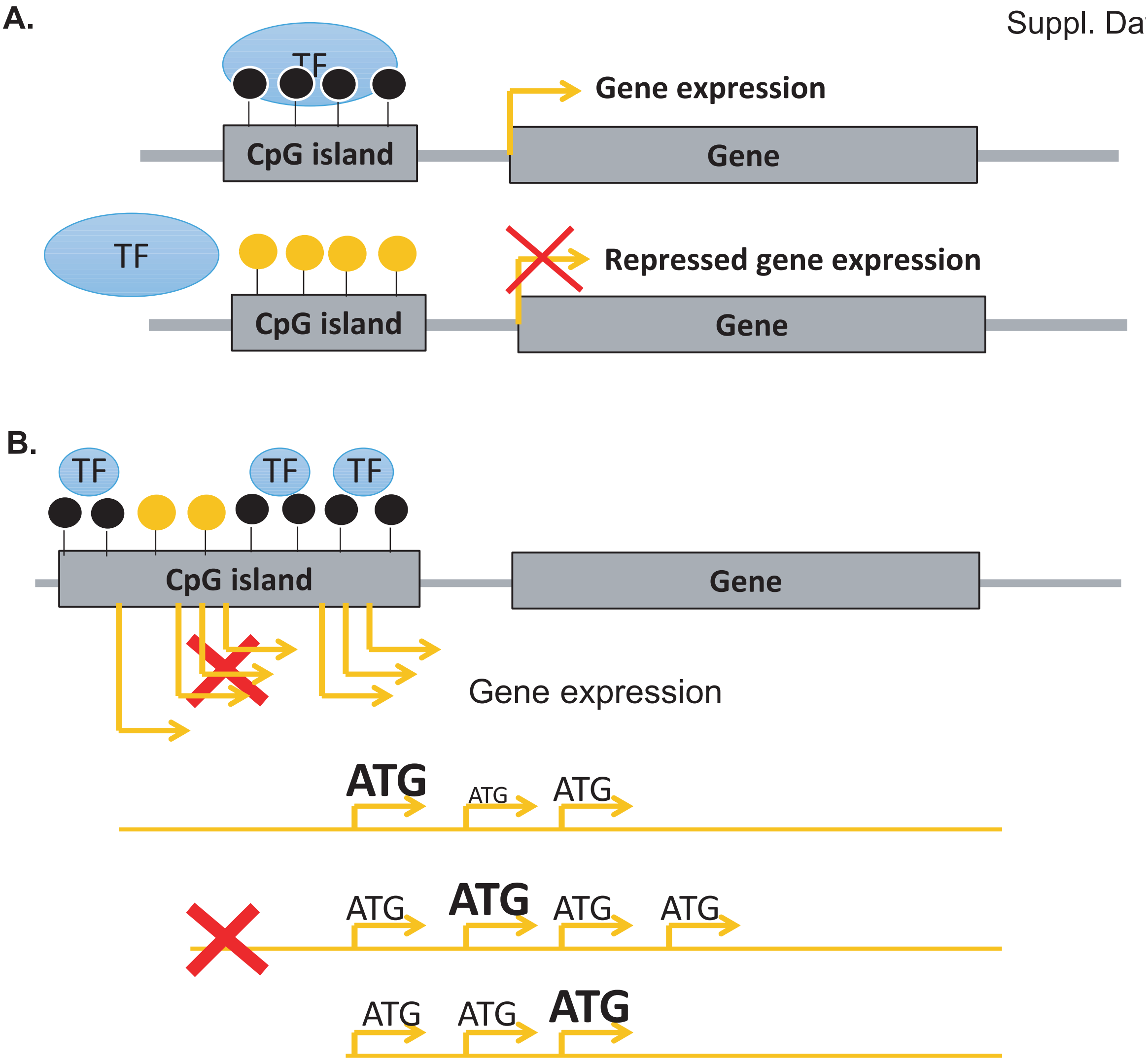

Supplementary Figure 17. Schematic representation of gene transcription and translation and how it is influenced by methylation. (A) Transcription process. Methylated CpG island stops the transcription factor (TF) from binding and blocks consequently the gene expression. (B) Translation process. Depending on where the transcription initiated the translation start codon within the mRNA differs.(Methylated: ; Non-Methylated)
